# Gemcitabine-Resistant Biomarkers in Bladder Cancer are Associated with Tumor-Immune Microenvironment

**DOI:** 10.3389/fcell.2021.809620

**Published:** 2022-01-21

**Authors:** Yuxuan Song, Yiqing Du, Caipeng Qin, Haohong Liang, Wenbo Yang, Jiaxing Lin, Mengting Ding, Jingli Han, Tao Xu

**Affiliations:** ^1^ Department of Urology, Peking University People’s Hospital, Beijing, China; ^2^ Biomedical Pioneering Innovation Center (BIOPIC), School of Life Sciences, Peking University, Beijing, China

**Keywords:** bladder cancer, gemcitabine, tumor immune microenvironment, GEO, TCGA

## Abstract

To identify key biomarkers in gemcitabine (GEM)-resistant bladder cancer (BCa) and investigate their associations with tumor-infiltrating immune cells in a tumor immune microenvironment, we performed the present study on the basis of large-scale sequencing data. Expression profiles from the Gene Expression Omnibus GSE77883 dataset and The Cancer Genome Atlas BLCA dataset were analyzed. Both BCa development and GEM-resistance were identified to be immune-related through evaluating tumor-infiltrating immune cells. Eighty-two DEGs were obtained to be related to GEM-resistance. Functional enrichment analysis demonstrated they were related to regulation of immune cells proliferation. Protein–protein interaction network selected six key genes (CAV1, COL6A2, FABP4, FBLN1, PCOLCE, and CSPG4). Immunohistochemistry confirmed the down-regulation of the six key genes in BCa. Survival analyses revealed the six key genes were significantly associated with BCa overall survival. Correlation analyses revealed the six key genes had high infiltration of most immune cells. Gene set enrichment analysis further detected the key genes might regulate GEM-resistance through immune response and drug metabolism of cytochrome P450. Next, microRNA-gene regulatory network identified three key microRNAs (hsa-miR-124-3p, hsa-miR-26b-5p, and hsa-miR-192-5p) involved in GEM-resistant BCa. Connectivity Map analysis identified histone deacetylase inhibitors might circumvent GEM-resistance. In conclusion, CAV1, COL6A2, FABP4, FBLN1, PCOLCE, and CSPG4 were identified to be critical biomarkers through regulating the immune cell infiltration in an immune microenvironment of GEM-resistance and could act as promising treatment targets for GEM-resistant muscle-invasive BCa.

## Introduction

Bladder cancer (BCa) is the ninth most prevalent cancer globally (Antoni et al., 2017; Bray et al., 2018). Approximately 540,000 new cases have been diagnosed and 195,000 patients die of BCa each year (Antoni et al., 2017; Bray et al., 2018). The BCa incidence is elevated worldwide and the tumor burden increases due to population aging and environmental pollution during the past 2 decades (Ebrahimi et al., 2019; Yang et al., 2019).

Although surgical operation has been utilized in BCa treatment, the prognosis is still poor (Kirkali et al., 2005; Goebell et al., 2008; Humphrey et al., 2016). The 5-year relapse rate after initial treatment is from 55% to 85% in non-muscle-invasive BCa (NMIBC) and the 5-year survival rate is from 30% to 45% in muscle-invasive BCa (MIBC) (Choueiri and Raghavan, 2008; Lightfoot et al., 2014; Antoni et al., 2017). Chemotherapy is a promising treatment for reducing the recurrence rate and improving the survival rate of BCa patients (Coen et al., 2019). Gemcitabine (GEM) is a kind of cytosine analogue that inhibits DNA synthesis. Combination therapy of GEM and other chemotherapeutic drugs has been widely utilized in the treatment of MIBC (Oh et al., 2009).

However, GEM-resistance causes a severe challenge in the treatment of MIBC. It is reported that the response rate of advanced MIBC with GEM treatment is less than 40%, which indicates a limited efficacy of GEM treatment (Kaufman et al., 2009; Sternberg et al., 2013). The long-term curative effects of GEM declined sharply with the extension of treatment time (Cao et al., 2018). In addition, inherent or acquired drug resistance is usually observed in clinical practice (Bergman et al., 2002; Wang and Lippard, 2005). As a consequence, it is necessary and vital to explore potential mechanisms of GEM-resistance. On the one hand, MIBC has the nature of high somatic-mutation frequency and molecular heterogeneity, which exerts a critical role in drug resistance (Giannopoulou et al., 2019). On the other hand, dysfunction of immune system exerts a crucial role in tumor resistance (Yu et al., 2018). Co-delivery of GEM and small interfering RNA targeting IDO1 could relieve the immune brakes and further alleviate the immune inhibition of M2 macrophages, which indicates that these immune cells are associated with regulation of immune response to GEM (Chen et al., 2020). In addition, bioinformatics analyses constructs a microRNA (miRNA)-gene regulatory network associated with alteration of memory CD4^+^ T cells in GEM-resistant pancreatic cancer cells, which suggests the immune system is implicated in the microenvironment of GEM-resistance (Gu et al., 2020).

The present study focused on the key genes, miRNA-gene regulatory network, and their immune microenvironment based on GEM-resistant BCa in order to explore reliable prognostic indicators and provide treatment targets for GEM-resistant BCa.

## Materials and methods

### The Cancer Genome Atlas (TCGA) Bladder Urothelial Carcinoma (BLCA) dataset

The gene expression profiling dataset and clinical data of TCGA BLCA (accessed September 1, 2021) were downloaded from the TCGA website (http://portal.gdc.cancer.gov/). TCGA BLCA comprised BCa tissues (*n* = 404) and adjacent normal tissues (*n* = 18). Among all samples, there were 18 pairs of BCa tissues (*n* = 18) and matched adjacent normal tissues (*n* = 18).

### GSE77883 dataset from Gene Expression Omnibus (GEO) database

Gene expression profiles of GSE77883 (accessed September 1st, 2021) were downloaded from GEO (http://www.ncbi.nlm.nih.gov/geo/). Six cells containing untreated T24 cells (*n* = 3) and GEM-resistant T24 cells (*n* = 3) were enrolled. RNA was extracted and measured through microarray (Platform: GPL17077 Agilent-039494 SurePrint G3 Human GE v2 8 × 60 K Microarray 039381).

### Gene Set Variation Analysis (GSVA)

We downloaded the gene set of TOOKER_GEMCITABINE_RESISTANCE_UP (M19654) (Tooker et al., 2007) from the Molecular Signatures Database (MSigDB: http://www.gsea-msigdb.org/gsea/index.jsp) (Liberzon et al., 2015), and M19654 is the key GEM-related gene set from chemical and genetic perturbations of MSigDB. The normalized GEM-resistance GSVA score of the gene set was measured for each BCa tissue from TCGA BLCA using the GSVA algorithm with the GSVA R package (Hänzelmann et al., 2013). The median value of the GEM-resistance score was used to divide all TCGA BCa tissues into high score of the GEM-resistance group (*n* = 202) and low score of the GEM-resistance group (*n* = 202).

### Immune cells analysis in tumor-immune microenvironment

Tumor-infiltrating immune cells were measured and analyzed with the MCPcounter (Microenvironment Cell Populations-counter) R package (Becht et al., 2016). In order to explore whether GEM-resistance is immune-related, we compared the immune cells between the high score of the GEM-resistance group (*n* = 202) and low score of the GEM-resistance group (*n* = 202) in TCGA BCa tissues. The Pearson method was adopted to assess the correlation between the normalized GEM-resistance GSVA score and immune cells. To further clarify the correlation between immune system and BCa development, we compared the immune cells between 18 pairs of BCa tissues (*n* = 18) and matched adjacent normal tissues (*n* = 18) in TCGA BLCA.

### Identification of differentially expressed genes (DEGs) in GSE77883 and TCGA BLCA datasets

Limma R package was adopted to detect DEGs between GEM-resistant T24 cells and untreated T24 cells in the GSE77883 dataset (Ritchie et al., 2015). In addition, we also identified DEGs between 18 pairs of BCa tissues and adjacent tissues in TCGA BLCA. Benjamini-Hochberg method (Benjamini and Hochberg, 1995; Benjamini and Hochberg, 2000) was used to adjust the p-values for multiplicity and control false discovery rate. The thresholds were |logFC| ≥ 1 and adjusted p-value (adj. p-value) < 0.05. Venn diagram was adopted to find overlapped DEGs in the GSE77883 and TCGA BLCA datasets. These overlapped DEGs were considered as GEM-resistant genes in BCa.

### Functional enrichment analysis

ClusterProfiler R package (Yu et al., 2012) was applied to identify the biological functions of overlapped DEGs through Gene Ontology (GO) and Kyoto Encyclopedia of Genes and Genomes (KEGG) pathway collections. Metascape (http://metascape.org) was utilized to identify the most closely enriched clusters (Zhou et al., 2019).

### Protein–protein interaction (PPI) network and selection of hub genes

We applied String (version 11.0: http://string-db.org/) (Szklarczyk et al., 2017) to construct interactions among proteins on the basis of the overlapped DEGs with the interaction score of 0.400 was set as threshold. In addition, cytoHubba in Cytoscape software screened 10 genes with highest connection degrees. Univariate Cox regression analysis further detected prognosis-related genes among the top 10 genes based on BCa patients (*n* = 404) from the TCGA BLCA dataset.

### Immunohistochemistry and validation by TCGA BLCA and Genotype-Tissue Expression (GTEx) datasets

Immunohistochemistry was extracted and analyzed from The Human Protein Atlas (THPA) database (http://www.proteinatlas.org/) (Uhlen et al., 2010). We evaluated expression levels of the identified six prognosis-related genes (CAV1, CSPG4, FBLN1, COL6A2, FABP4, and PCOLCE) between tumor and normal tissues at protein level.

To confirm the differential expression of the six prognosis-related genes in a larger sample size, box plots were adopted to compare the expression of CAV1, CSPG4, FBLN1, COL6A2, FABP4, and PCOLCE between BCa tissues and normal tissues from the TCGA BLCA dataset and the GTEx dataset through Gene Expression Profiling Interactive Analysis (GEPIA) (http://gepia.cancer-pku.cn/) (Tang et al., 2017).

### Survival analysis

Based on the TCGA BLCA dataset, we analyzed the six selected genes (CAV1, CSPG4, FBLN1, COL6A2, FABP4, and PCOLCE) with overall survival (OS) and disease-free survival (DFS) through the Kaplan–Meier (KM) Plotter (http://kmplot.com/analysis/) (Nagy et al., 2018).

### Predictive value of the six hub genes in immunotherapy

CAMOIP (Comprehensive Analysis on Multi-Omics of Immunotherapy in Pan-cancer) is a tool for analyzing the expression data and mutation data from the immunotherapy-treated projects, using a standard processing pipeline (Lin et al., 2021). The IMvigor210 cohort to investigate the clinical activity of immunotherapy with atezolizumab in metastatic BCa was used for an integrated biomarker evaluation (Mariathasan et al., 2018). We used gene expression profiling from the IMvigor210 cohort to evaluate the predictive value of the six key genes (CAV1, COL6A2, FABP4, FBLN1, PCOLCE, and CSPG4) in OS after immunotherapy through CAMOIP.

### Pearson correlation analysis explored the six hub genes with tumor-infiltrating immune cells

TIMER (Tumor Immune Estimation Resource) (http://timer.cistrome.org/) (Li et al., 2016; Li et al., 2017) was adopted to measure the impacts of immune cells on BCa OS through separating all BCa samples (*n* = 404) into high and low abundance groups based on median value of each immune cell abundance. In addition, Pearson method measured correlations between immune cells and expression levels of the six genes (CAV1, CSPG4, FBLN1, COL6A2, FABP4, and PCOLCE) in BCa patients (*n* = 404) from the TCGA BLCA dataset through Pearson correlation analysis.

### Genetic mutation analysis

The cBioPortal database (http://www.cbioportal.org/) was utilized to investigate mutations of the six hub genes (CAV1, CSPG4, FBLN1, COL6A2, FABP4, and PCOLCE) in BCa patients (*n* = 404) from the TCGA BLCA dataset. Survival analysis and immune cells were also explored based on genetic mutations.

### miRNA-Gene regulatory network

Two databases including miRTarbase (http://mirtarbase.mbc.nctu.edu.tw/php/) (Chou et al., 2018) and Targetscan (http://www.targetscan.org/vert_72/) (Agarwal et al., 2015) were experimentally validated miRNA-target gene interaction databases and they were adopted to predict upstream miRNAs and to build the miRNA-gene regulatory network. Venn diagram was used to identify overlapped miRNAs as key miRNAs and KM Plotter was utilized to evaluate the effects of key miRNAs on BCa OS based on BCa patients from the TCGA BLCA dataset as mentioned before. Pearson method was performed to evaluate the pairwise gene correlation in BCa samples from the TCGA BLCA dataset.

### Gene set enrichment analysis (GSEA)

To clarify the roles of the six hub genes (CAV1, CSPG4, FBLN1, COL6A2, FABP4, and PCOLCE), we applied GSEA to analyze the enrichment of BCa samples (n = 404) in the TCGA BLCA dataset by assessing the normalized enrichment score (NES) (Subramanian et al., 2007).

### Screening of potential targeted drugs in GEM-resistant BCa

The Connectivity Map (CMAP) database (http://portals.broadinstitute.org/cmap) (Lamb et al., 2006; Lamb, 2007) was utilized to explore the potential drugs with antagonism or synergism to GEM-resistance. This database used details of DEGs to identify potential targeted drugs. Eighty-two key DEGs in GEM-resistant BCa were uploaded to the CMAP database. Next, the 82 key DEGs were compared to expression profiles stored in CMAP in order to select potential drugs. Drugs with p-value <0.05 were considered as significant targeted drugs to GEM-resistance.

### Statistical analysis

Statistical analyses were performed by R software (v3.6.1: http://www.r-project.org), GraphPad Prism 7.0, Metascape, GEPIA, KM Plotter, TIMER, and cBioPortal. Univariate Cox regression, KM method, and log-rank test were adopted for survival analysis by calculating hazard ratio (HR) and 95% confidence interval (CI). Student’s *t* test was applied to evaluate quantitative variables. The correlation coefficient, *R*-value, was used to estimate the strength of Pearson correlation analysis. The two-sided p-value <0.05 was set as the threshold. Cytoscape software (v.3.6.1) was adopted for visualization of networks.

## Results

**FIGURE 1:**
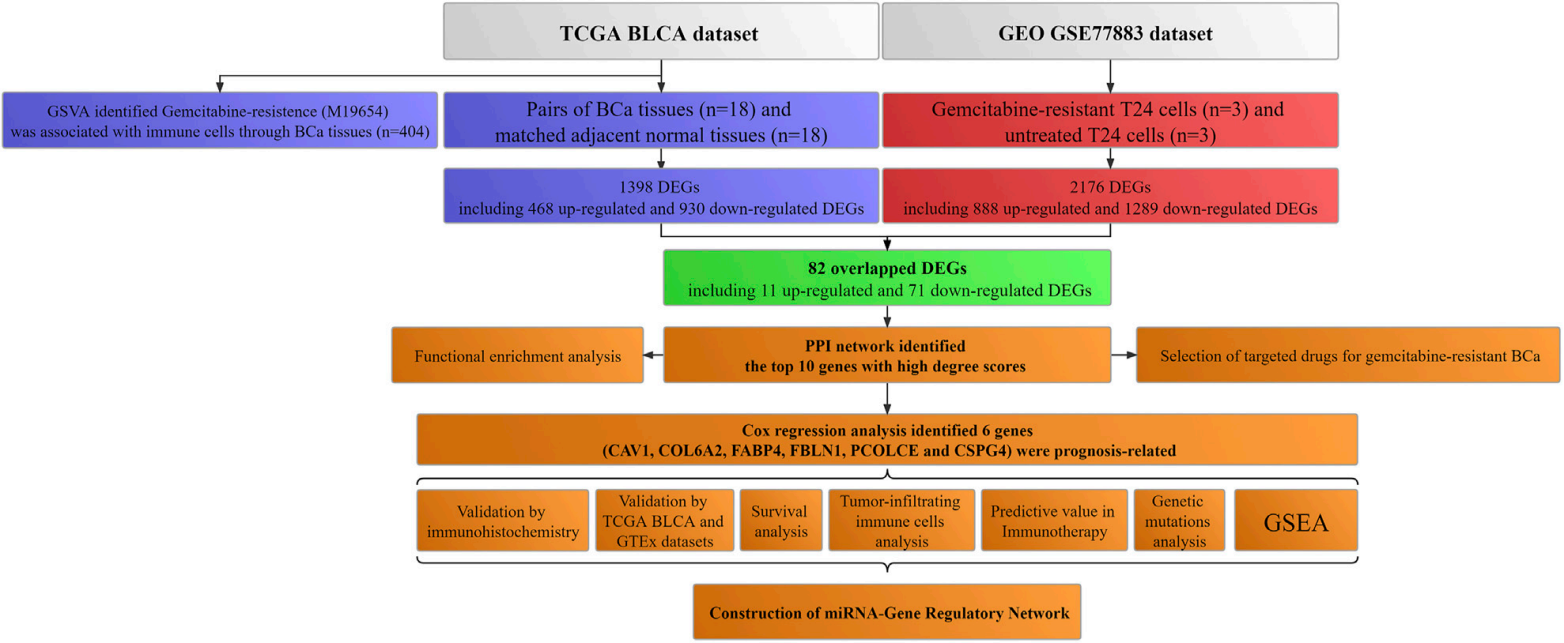
Workflow of this study. TCGA: The Cancer Genome Atlas; BLCA: Bladder urothelial carcinoma; GEO: Gene Expression Omnibus; BCa: Bladder cancer; GSVA: Gene set variation analysis; GTEx: Genotype-tissue expression; DEGs: Differentially expressed genes; PPI: Protein–protein interaction; miRNA: microRNA; GSEA: Gene set enrichment analysis.

Figure 1 showed the workflow.

### Tumor immune microenvironment in GEM-resistance

**FIGURE 2:**
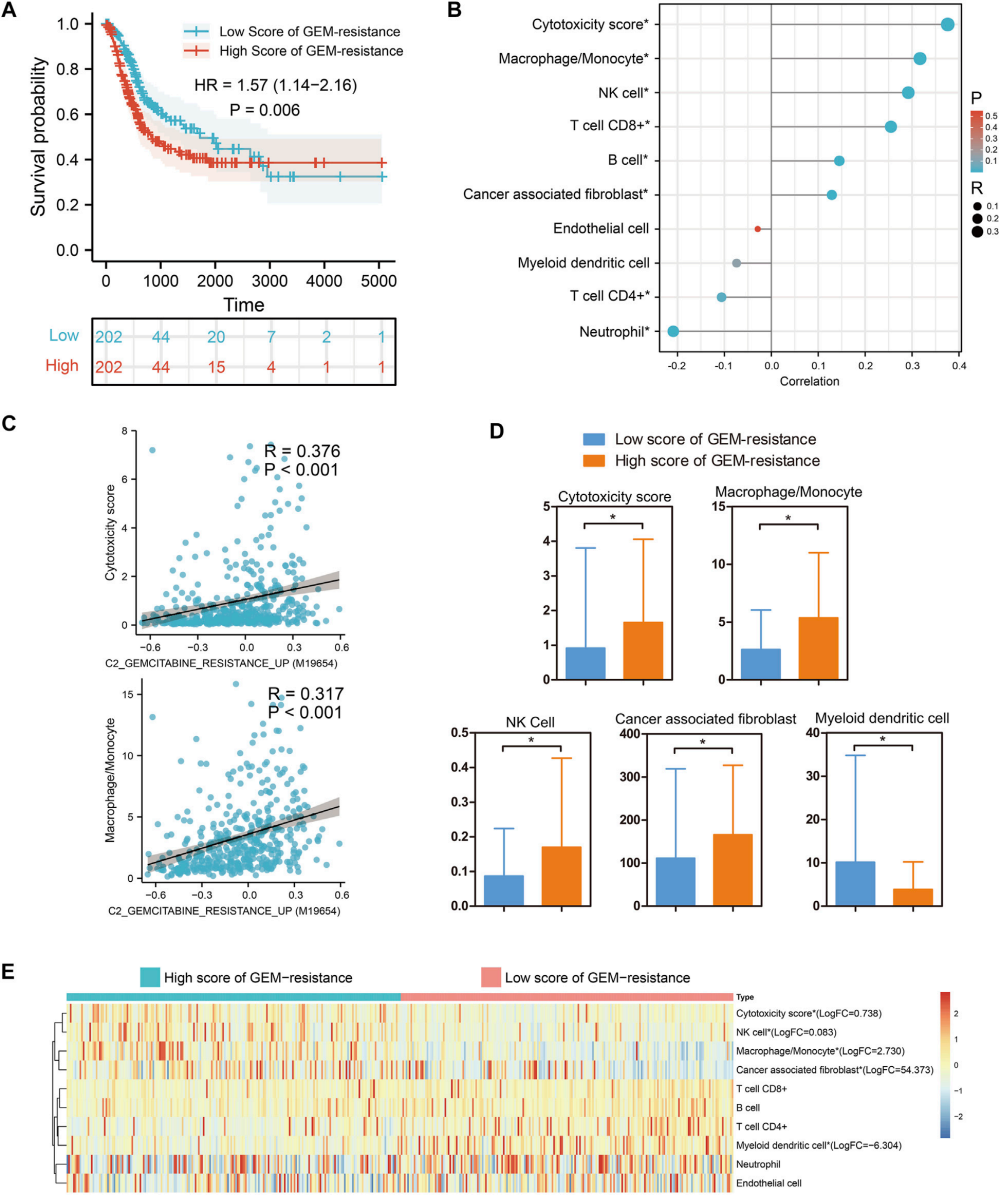
Gene set variation analysis identified that gemcitabine (GEM)-resistance was associated with prognosis and immune microenvironment in 404 bladder cancer (BCa) patients from the TCGA BLCA dataset. **(A)** Kaplan–Meier survival indicated BCa patients with high score of GEM-resistance had poor overall survival; **(B,C)** Correlations between GEM-resistance score and immune cells; **(D,E)** Differences in abundance of immune cells between high score and low score of GEM-resistance. *p < 0.05.

Based on 404 BCa tissues from the TCGA BLCA dataset, GSVA analysis indicated that BCa patients with high score of GEM-resistance had worse OS compared with those with low score (HR = 1.57, 95%CI = 1.14–2.16) (p = 0.006) (Figure 2A). Furthermore, correlation analysis revealed GEM-resistance score was positively associated with cytotoxicity scores (R = 0.376, p < 0.001), macrophages/monocytes (R = 0.317, p < 0.001), NK cells, and cancer-associated fibroblasts. GEM-resistance score was negatively associated with myeloid dendritic cells (Figures 2B,C). In addition, BCa patients with high score of GEM-resistance had higher abundance of the cytotoxicity scores, macrophages/monocytes, NK cells, and cancer-associated fibroblasts as well as lower abundance of myeloid dendritic cells in comparison with those with low score, which confirmed the results of correlation analysis (Figures 2D,E). From the above results, we identified that GEM-resistance score in BCa is closely related to immune microenvironment. In addition, GSVA analysis based on the GSE77883 dataset indicated that GEM-resistant T24 cells had higher score of GEM-resistance than untreated T24 cells (p = 0.03) (Supplementary Figure S1).

### Tumor-immune microenvironment in BCa development

Based on 18 pairs of BCa tissues and matched adjacent normal tissues, immune cells infiltration analysis suggested that the abundance of B cells, myeloid dendritic cells, endothelial cells, and cancer associated fibroblast cells was obviously down-regulated in BCa tissues compared with matched adjacent normal tissues (p < 0.05). However, for other immune cells, no change was observed (p > 0.05) (Supplementary Figure S2). From the above results, we identified that BCa development is closely related to an immune microenvironment.

### Identification of key genes in GSE77883 and TCGA BLCA datasets

**FIGURE 3:**
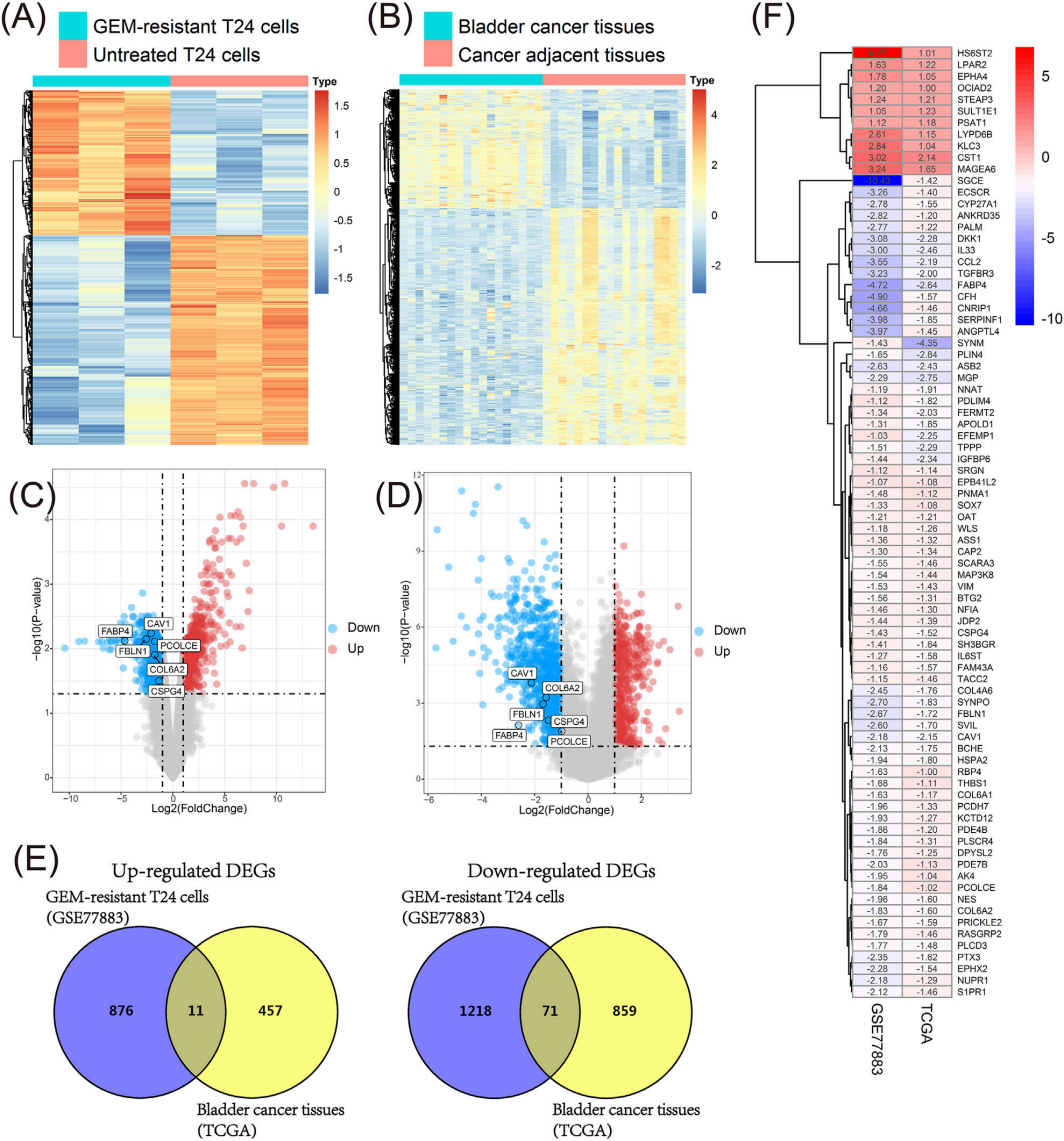
Identification of key genes in the GSE77883 and TCGA BLCA datasets. **(A)** Heat map of differentially expressed genes (DEGs) between gemcitabine (GEM)-resistant T24 cells and untreated T24 cells based on the GSE77883 dataset; **(B)** Heat map of DEGs between bladder cancer (BCa) tissues and matched adjacent normal tissues based on the TCGA BLCA dataset; **(C)** Volcano plot of DEGs between GEM-resistant T24 cells and untreated T24 cells based on the GSE77883 dataset; **(D)** Volcano plot of DEGs between BCa tissues and matched adjacent normal tissues based on the TCGA BLCA dataset; **(E)** Venn diagram identified overlapped DEGs in both GSE77883 and TCGA BLCA datasets; **(F)** Heat map of 82 overlapped DEGs and they were key genes in both GEM-resistance and BCa development. adj.P.-value was adjusted p-value for Benjamini-Hochberg (BH) method.

We firstly compared GEM-resistant T24 cells with untreated T24 cells in the GSE77883 dataset; 2,176 DEGs containing 888 up-regulated and 1,289 down-regulated genes were obtained in GEM-resistant BCa cells (Figure 3A and Figure 3C).

In addition, we compared 18 pairs of BCa tissues and matched adjacent normal tissues in the TCGA BLCA dataset. We obtained 1,398 DEGs containing 468 up-regulated and 930 down-regulated genes in BCa tissues (Figure 3B and Figure 3D).

In order to investigate which genes were associated with both GEM-resistance and BCa development, Venn diagram combined the DEGs from two datasets and identified the overlapped DEGs obtained in both datasets. Ultimately, 82 overlapped DEGs (11 overlapped up-regulated and 71 overlapped down-regulated genes) were obtained and were considered as GEM-resistant genes in BCa (Figures 3E,F) (Supplementary Table S1).

### Functional enrichment analysis

**FIGURE 4:**
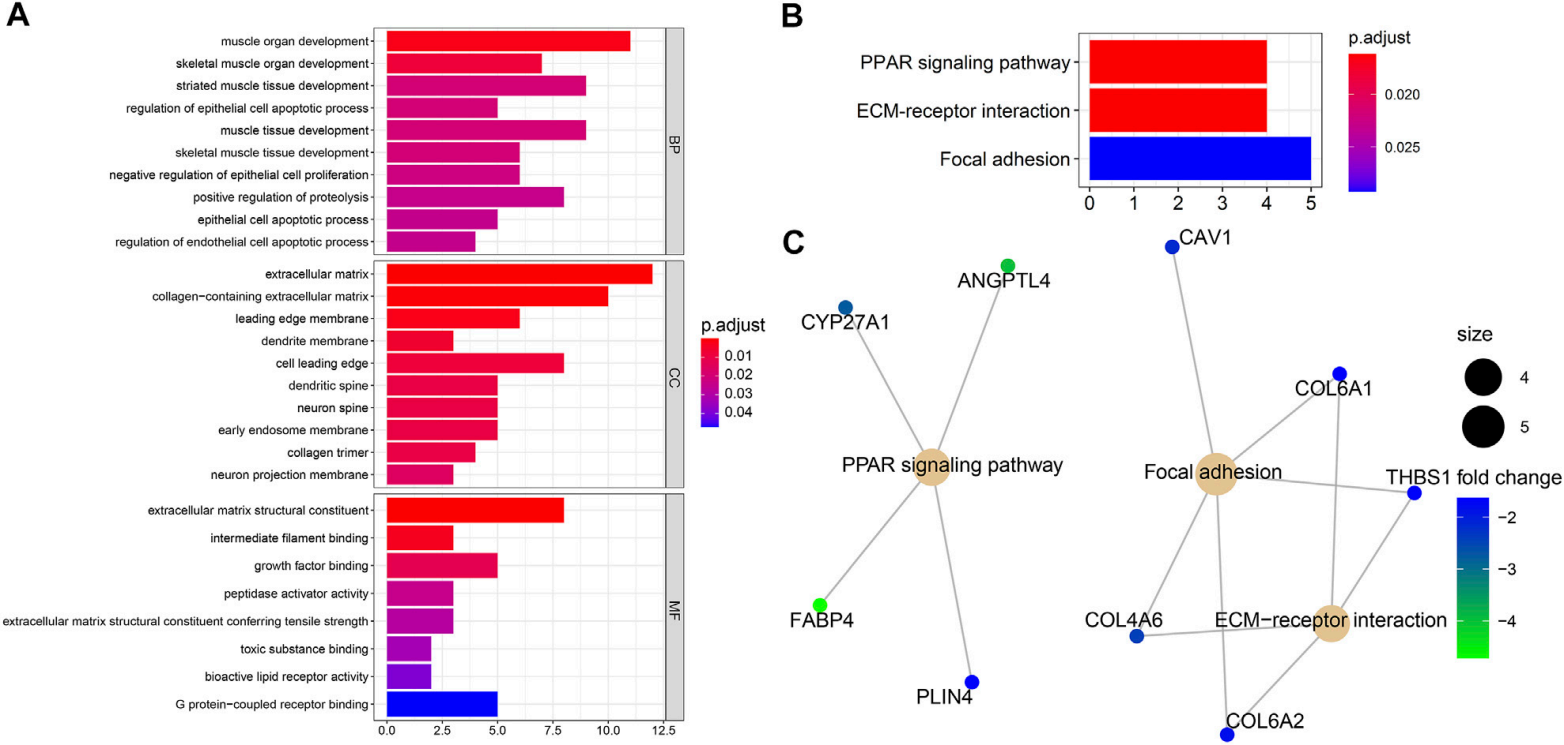
Enrichment analyses through 82 overlapped differentially expressed genes. **(A)** Gene ontology enrichment analysis. Biological process (BP) indicated they were enriched in regulation of epithelial cell apoptotic process (GO:1904035) and muscle tissue development (GO:0060537); cell component (CC) indicated they were enriched in extracellular matrix (GO:0031012) and cell leading edge (GO:0031252); molecular function (MF) indicated they were enriched in growth factor binding (GO:0019838) and toxic substance binding (GO:0015643); **(B,C)** Kyoto Encyclopedia of Genes and Genomes (KEGG) pathway and gene-concept network analysis. They were enriched in the peroxisome proliferator-activated receptor (PPAR) signaling pathway (hsa03320), extracellular matrix (ECM)-receptor interaction (hsa04512), and focal adhesion (hsa04510).

Biological process analysis indicated that the 82 overlapped DEGs were enriched in regulation of the epithelial cell apoptotic process (GO:1904035) and muscle tissue development (GO:0060537). Component analysis detected that they were mainly located at the extracellular matrix (GO:0031012) and cell leading edge (GO:0031252). Molecular function analysis demonstrated that they participated in growth factor binding (GO:0019838) and toxic substance binding (GO:0015643) (Figure 4A and Table 1). Pathway analyses identified GEM-resistance was associated with the peroxisome proliferator-activated receptor (PPAR) signaling pathway (hsa03320), extracellular matrix (ECM)-receptor interaction (hsa04512), and focal adhesion (hsa04510) (Figures 4B,C and Table 1).

**TABLE 1:** Functional enrichment analysis results

Term	Description	Category	Adjusted p-value
GO:0007517	Muscle organ development	GO (BP)	2.59E-03
GO:0060538	Skeletal muscle organ development	GO (BP)	7.46E-03
GO:0014706	Striated muscle tissue development	GO (BP)	1.99E-02
GO:1904035	Regulation of epithelial cell apoptotic process	GO (BP)	1.99E-02
GO:0060537	Muscle tissue development	GO (BP)	1.99E-02
GO:0007519	Skeletal muscle tissue development	GO (BP)	2.00E-02
GO:0050680	Negative regulation of epithelial cell proliferation	GO (BP)	2.18E-02
GO:0045862	Positive regulation of proteolysis	GO (BP)	2`.45E-02
GO:1904019	Epithelial cell apoptotic process	GO (BP)	2.49E-02
GO:2000351	Regulation of endothelial cell apoptotic process	GO (BP)	2.49E-02
GO:0031012	Extracellular matrix	GO (CC)	1.04E-04
GO:0062023	Collagen-containing extracellular matrix	GO (CC)	6.16E-04
GO:0031256	Leading edge membrane	GO (CC)	2.91E-03
GO:0032590	Dendrite membrane	GO (CC)	5.90E-03
GO:0031252	Cell leading edge	GO (CC)	8.01E-03
GO:0043197	Dendritic spine	GO (CC)	9.77E-03
GO:0044309	Neuron spine	GO (CC)	9.77E-03
GO:0031901	Early endosome membrane	GO (CC)	9.77E-03
GO:0005581	Collagen trimer	GO (CC)	1.03E-02
GO:0032589	Neuron projection membrane	GO (CC)	1.55E-02
GO:0005201	Extracellular matrix structural constituent	GO (MF)	7.61E-05
GO:0019215	Intermediate filament binding	GO (MF)	3.35E-03
GO:0019838	Growth factor binding	GO (MF)	1.15E-02
GO:0016504	Peptidase activator activity	GO (MF)	2.46E-02
GO:0030020	Extracellular matrix structural constituent conferring tensile strength	GO (MF)	2.90E-02
GO:0015643	Toxic substance binding	GO (MF)	3.26E-02
GO:0045125	Bioactive lipid receptor activity	GO (MF)	3.95E-02
GO:0001664	G protein-coupled receptor binding	GO (MF)	4.73E-02
hsa03320	Peroxisome proliferators-activated receptor (PPAR) signaling pathway	KEGG pathway	1.62E-02
hsa04512	Extracellular matrix (ECM)-receptor interaction	KEGG pathway	1.62E-02
hsa04510	Focal adhesion	KEGG pathway	2.93E-02

GO: Gene Ontology; KEGG: Kyoto Encyclopedia of Genes and Genomes; BP: biological process; CC: cell component; MF: molecular function.

Metascape identified the interactions of the main 19 clustered enrichment terms (Supplementary Figure S3 and Supplementary Table S2). Table 2 shows that negative regulation of cell proliferation (GO:0008285) and its relevant enrichment terms were significantly associated with immune cells including endothelial cells (GO:2000351), T cells (GO:0050870), and granulocytes (GO:0071621), which confirmed the close relationship between GEM-resistance and immune system.

**TABLE 2:** Immune-related enrichment terms associated with immune cells proliferation

Term	Description	Category	Adjusted p-value
GO:0008285	Negative regulation of cell proliferation	GO (BP)	1.13E-05
GO:2000351	Regulation of endothelial cell apoptotic process	GO (BP)	5.92E-05
GO:0072577	Endothelial cell apoptotic process	GO (BP)	8.03E-05
GO:2000353	Positive regulation of endothelial cell apoptotic process	GO (BP)	8.22E-05
GO:0002696	Positive regulation of leukocyte activation	GO (BP)	2.15E-03
GO:0001937	Negative regulation of endothelial cell proliferation	GO (BP)	2.48E-03
GO:1903037	Regulation of leukocyte cell-cell adhesion	GO (BP)	4.40E-03
GO:0050870	Positive regulation of T cell activation	GO (BP)	5.35E-03
GO:0007159	Leukocyte cell-cell adhesion	GO (BP)	6.74E-03
GO:0030595	Leukocyte chemotaxis	GO (BP)	7.05E-03
GO:0050900	Leukocyte migration	GO (BP)	7.37E-03
GO:1903039	Positive regulation of leukocyte cell-cell adhesion	GO (BP)	7.39E-03
GO:0071621	Granulocyte chemotaxis	GO (BP)	7.78E-03

GO: Gene Ontology; BP: biological process.

### PPI network and selection of hub genes

**FIGURE 5:**
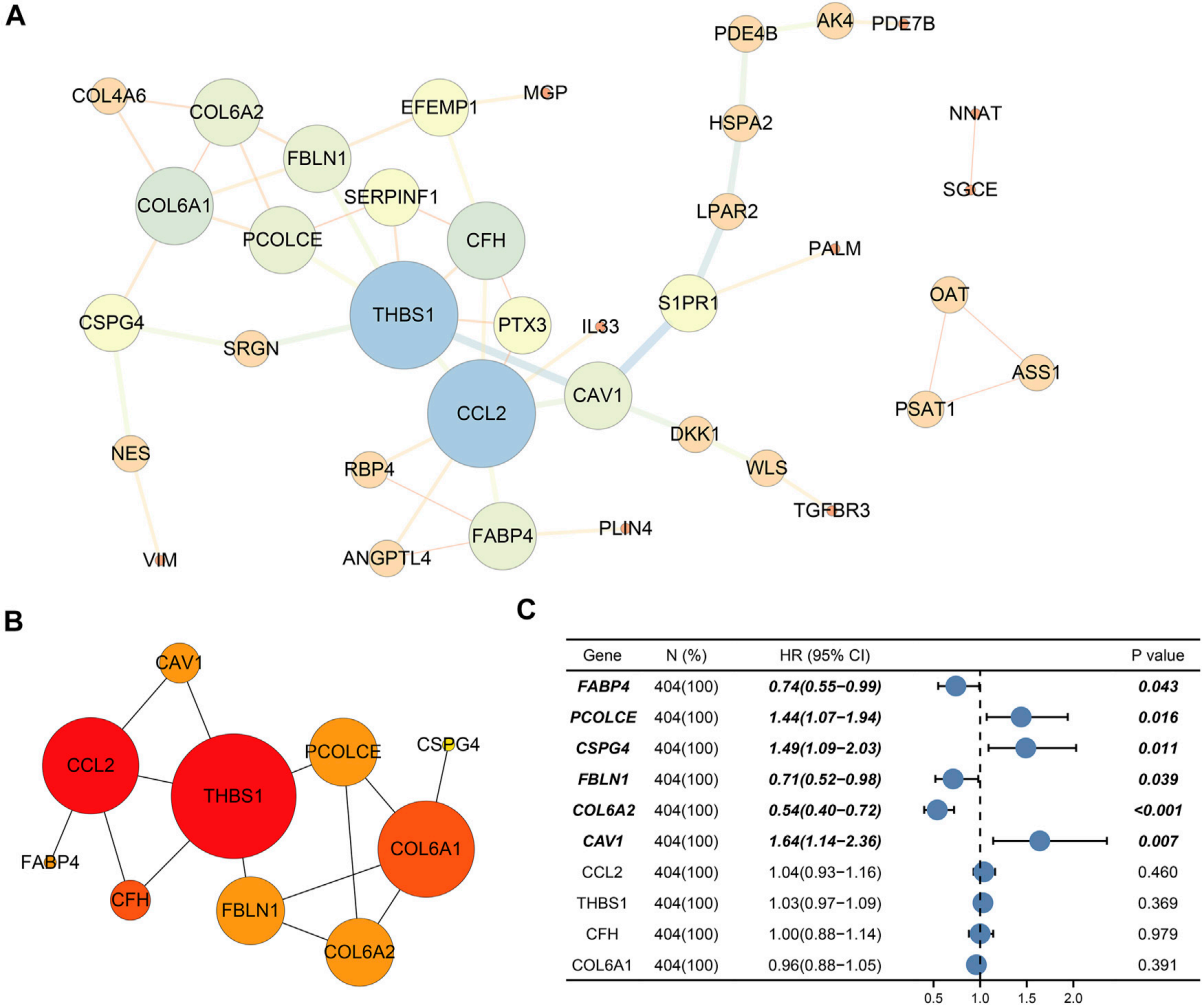
Protein–protein interaction (PPI) network and selection of hub genes. **(A)** PPI network of DEGs; **(B)** Hub module; **(C)** Six prognostic genes (CAV1, COL6A2, FABP4, FBLN1, PCOLCE, and CSPG4) through univariate Cox regression. Bold genes meant prognosis-related genes.

Finally, PPI network enrolled 37 nodes and 49 edges (Figure 5A). Among the interactions,CAV1, COL6A2, FABP4, FBLN1, PCOLCE, CSPG4, CCL2, THBS1, CFH, and COL6A1 with the highest degree scores were considered as the top 10 genes. Figure 5B showed the key module constituted by the 10 genes.

Ultimately, univariate Cox regression analysis detected that six genes (CAV1, COL6A2, FABP4, FBLN1, PCOLCE, and CSPG4) were identified to be prognosis-related (p < 0.05) (Table 3) (Figure 5C).

**TABLE 3:** The six hub genes with high degree scores

Gene symbol	Ensembel ID	Description	Type	Hazard ratio (95% confidence interval)	p-Value
CAV1	ENSG00000105974	Caveolin 1	Down-regulated	1.64 (1.14–2.36)	0.007
COL6A2	ENSG00000142173	Collagen type VI alpha 2 chain	Down-regulated	0.54 (0.40–0.72)	<0.001
FABP4	ENSG00000170323	Fatty acid binding protein 4	Down-regulated	0.74 (0.55–0.99)	0.043
FBLN1	ENSG00000077942	Fibulin 1	Down-regulated	0.71 (0.52–0.98)	0.039
PCOLCE	ENSG00000106333	Procollagen C-endopeptidase enhancer	Down-regulated	1.44 (1.07–1.94)	0.016
CSPG4	ENSG00000173546	Chondroitin sulfate proteoglycan 4	Down-regulated	1.49 (1.09–2.03)	0.011

### Immunohistochemistry and validation of hub genes by TCGA and GTEx datasets

**FIGURE 6:**
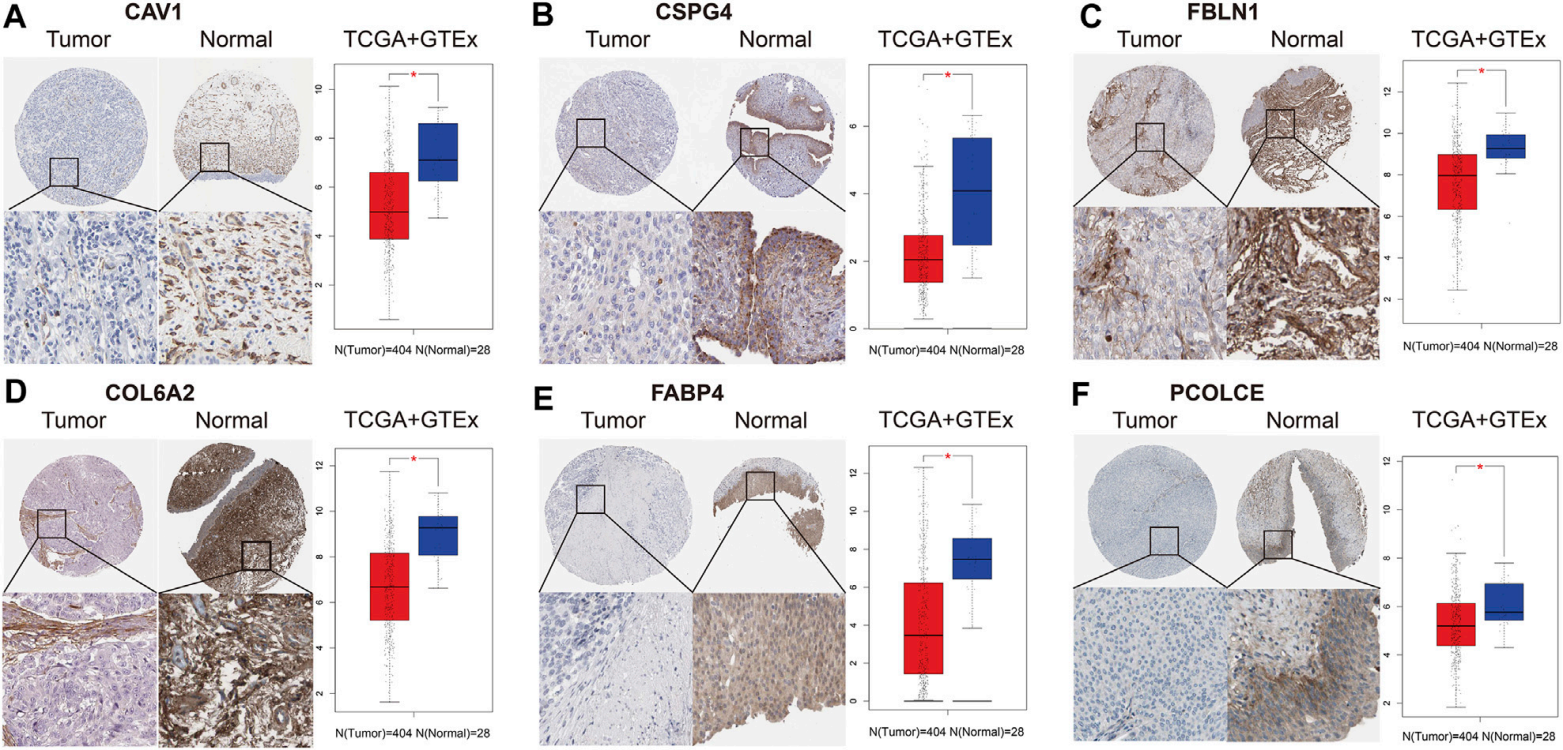
Immunohistochemistry and validation of six hub genes by the TCGA BLCA and GTEx datasets. Immunohistochemistry from THPA (left part in each subfigure) and bladder cancer (BCa) tissues (*n* = 404) and normal tissues (*n* = 28) from the TCGA BLCA and GTEx datasets (right part in each subfigure) indicated that the six selected genes (CAV1, COL6A2, FABP4, FBLN1, PCOLCE, and CSPG4) were down-regulated in BCa. **(A)** CAV1; **(B)** CSPG4; **(C)** FBLN1; **(D)** COL6A2; **(E)** FABP4; **(F)** PCOLCE.

We collected and analyzed immunohistochemistry of BCa tissues and normal bladder tissues from THPA. Expression levels of the six hub genes (CAV1, COL6A2, FABP4, FBLN1, PCOLCE, and CSPG4) were evaluated at protein level. Figure 6 reveals the six hub genes were down-regulated in BCa tissues.

In addition, we also validated the six hub genes between 404 BCa tissues and 28 normal bladder tissues from the TCGA BLCA and GTEx datasets. Box plots indicated that they were all down-regulated in BCa samples (p < 0.05), which was in accord with the results of immunohistochemistry (Figure 6).

### Survival analysis and predictive value of the six hub genes in immunotherapy

KM curves suggested that the six hub genes could influence the OS time (Supplementary Figure S4). Lower expression levels of FABP4, FBLN1, and COL6A2 indicated worse OS time (p < 0.05). However, higher expression levels of CSPG4, CAV1, and PCOLCE might be indicators for worse OS time (p < 0.05) for BCa patients. In addition, we identified that FABP4, CSPG4, COL6A2, and PCOLCE were related to DFS time of BCa (p < 0.05) (Supplementary Figure S4).

**FIGURE 7:**
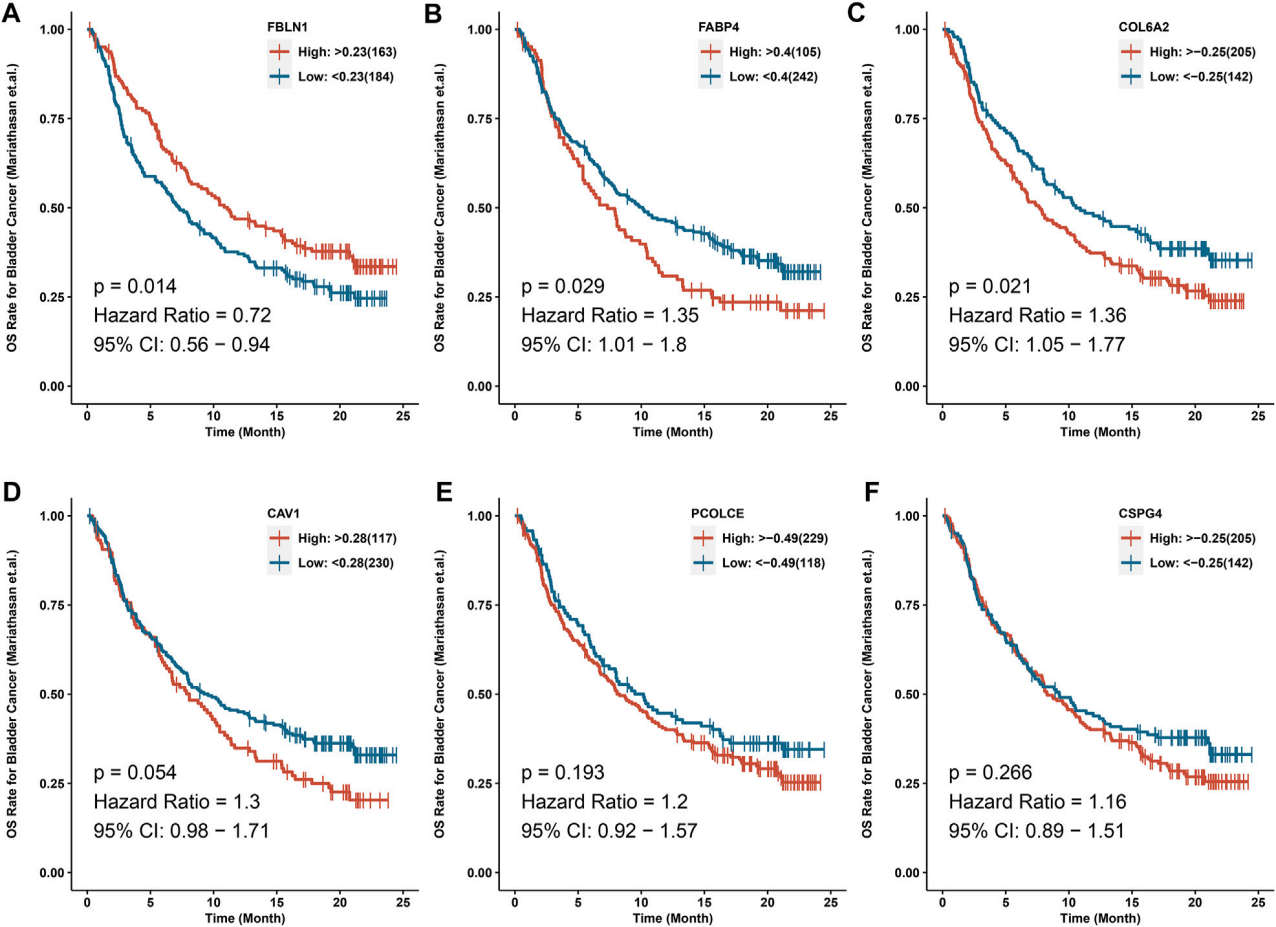
IMvigor210 cohort indicated that COL6A2, FABP4 and FBLN1 could predict the OS after immunotherapy with atezolizumab. **(A)** FBLN1; **(B)** FABP4; **(C)** COL6A2; **(D)** CAV1; **(E)** PCOLCE; **(F)** CSPG4.

IMvigor210 cohort indicated that COL6A2 (HR > 1), FABP4 (HR > 1), and FBLN1 (HR < 1) could predict the OS after immunotherapy with atezolizumab (p < 0.05) (Figure 7).

### Tumor-infiltrating immune cells with hub genes in TCGA BLCA dataset

We used TIMER to clarify the effects of immune cells on OS of BCa patients in the TCGA BLCA dataset. We found that five immune cells (CD4^+^ T cells, CD8^+^ T cells, neutrophils, endothelial cells, and cancer associated fibroblast cells) were associated with OS of BCa. Patients with high abundance of CD4^+^ T cells and CD8^+^ T cells had longer OS time than those with low infiltration levels. However, patients with low infiltration levels of neutrophils, endothelial cells, and cancer-associated fibroblast cells had longer OS time compared with those with high infiltration levels of these immune cells (Supplementary Figure S5).

**FIGURE 8:**
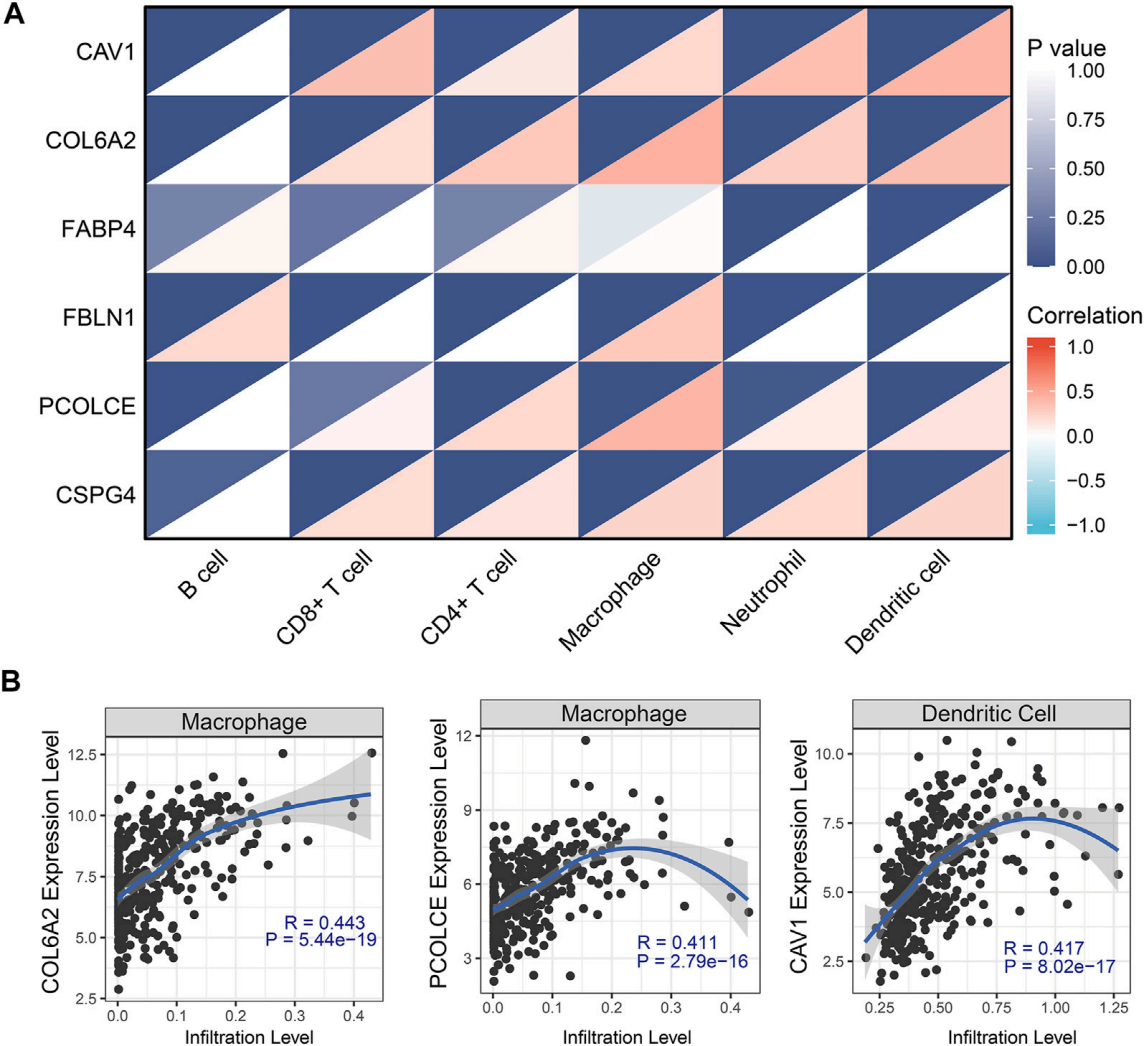
Tumor-infiltrating immune cell analysis with the six hub genes (CAV1, COL6A2, FABP4, FBLN1, PCOLCE, and CSPG4) through Pearson correlation method. **(A)** Heat map showed correlations between immune cells and six hub genes; **(B)** Robust correlations (R-value more than 0.400) was identified between CAV1, COL6A2, and PCOLCE and immune cells.

Pearson correlation analysis evaluated the correlations between the six hub genes (CAV1, COL6A2, FABP4, FBLN1, PCOLCE, and CSPG4) and abundance of main immune cells (Table 4 and Figure 8A) (Supplementary Figure S6). When we restricted the robust R-value to more than 0.400, we found CAV1 had strong correlation with dendritic cells. COL6A2 and PCOLCE had strong correlations with macrophages (Figure 8B).

**TABLE 4:** Pearson correlation analysis indicated the six hub genes (CAV1, COL6A2, FABP4, FBLN1, PCOLCE and CSPG4) were associated with immune cells infiltration

Immune cells	Hub gene	R-value	p-Value
B cell	CAV1	−0.193	**2.22E−04**
—	COL6A2	−0.18	**5.87E−04**
—	FABP4	0.053	3.09E−01
—	FBLN1	0.224	**1.58E−05**
—	PCOLCE	−0.146	**5.34E−03**
—	CSPG4	−0.087	9.81E−02
CD8+ T cell	CAV1	0.356	**2.24E−12**
—	COL6A2	0.198	**1.35E−04**
—	FABP4	−0.065	2.18E−01
—	FBLN1	−0.133	**1.09E−02**
—	PCOLCE	0.062	2.37E−01
—	CSPG4	0.198	**1.35E−04**
CD4+ T cell	CAV1	0.127	**1.55E−02**
—	COL6A2	0.303	**3.48E−09**
—	FABP4	0.054	3.04E−01
—	FBLN1	−0.188	**3.13E−04**
—	PCOLCE	0.215	**3.42E−05**
—	CSPG4	0.16	**2.21E−03**
Macrophage	CAV1	0.217	**2.99E−05**
—	COL6A2	**0.443**	**5.44E−19**
—	FABP4	0.008	8.75E−01
—	FBLN1	0.305	**2.61E−09**
—	PCOLCE	**0.411**	**2.79E−16**
—	CSPG4	0.24	**3.39E−06**
Neutrophil	CAV1	0.348	**9.07E−12**
—	COL6A2	0.271	**1.52E−07**
—	FABP4	−0.159	**2.40E−03**
—	FBLN1	−0.141	**6.94E−03**
—	PCOLCE	0.102	5.28E−02
—	CSPG4	0.21	**5.62E−05**
Dendritic cell	CAV1	**0.417**	**8.02E−17**
—	COL6A2	0.368	**3.71E−13**
—	FABP4	−0.127	**1.50E−02**
—	FBLN1	−0.262	**3.92E−07**
—	PCOLCE	0.156	**2.82E−03**
—	CSPG4	0.235	**5.85E−06**

The bold values indicated statistical significance.

**FIGURE 9:**
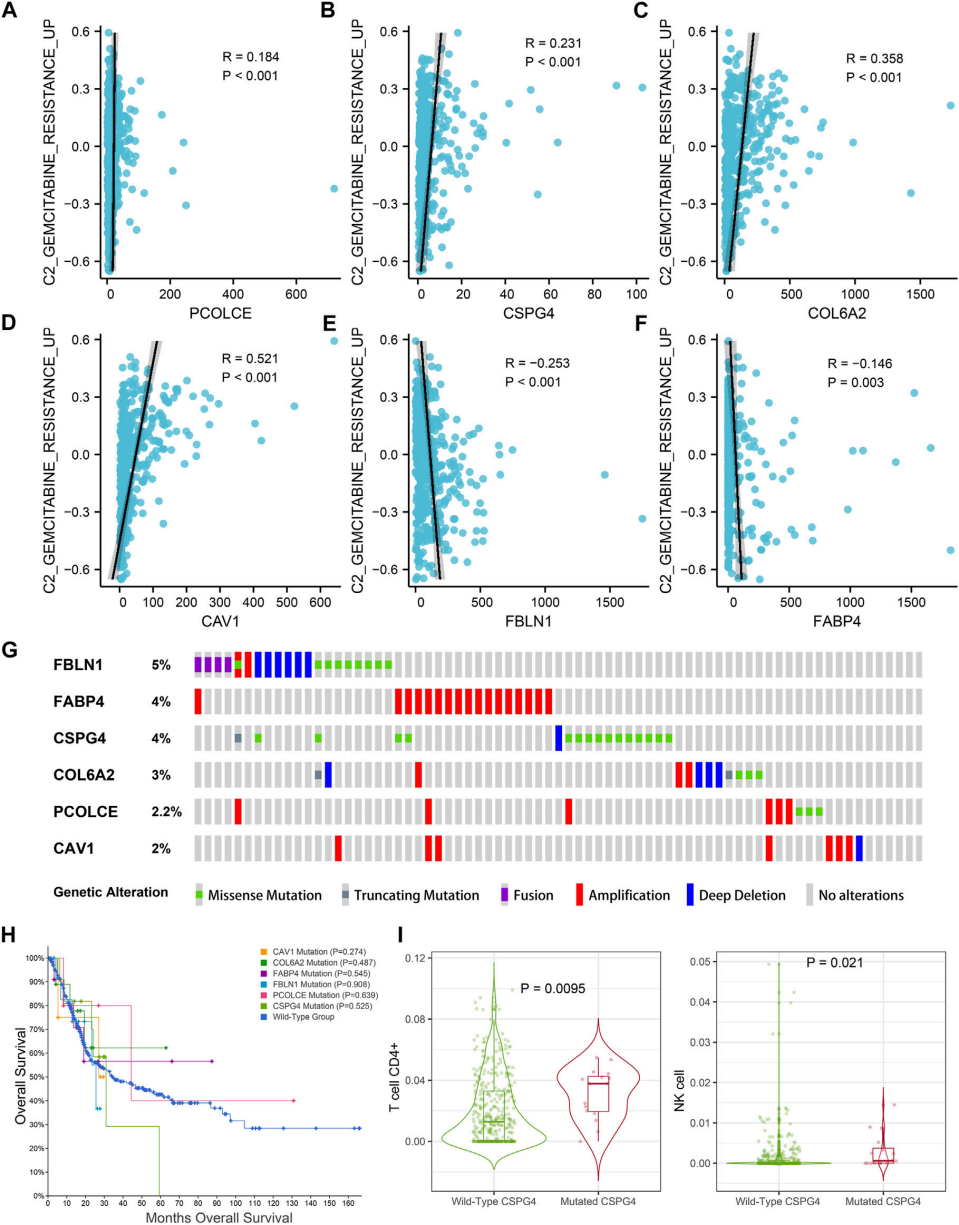
Genetic mutations analysis of the six hub genes (CAV1, COL6A2, FABP4, FBLN1, PCOLCE, and CSPG4) in the TCGA BLCA dataset. **(A–F)** Correlations between GEM-resistance score and the six hub genes; **(G)** Mutation frequencies of CAV1, COL6A2, FABP4, FBLN1, PCOLCE, and CSPG4 in the TCGA BLCA dataset; **(H)** Kaplan–Meier survival curves showed that genetic mutations of six selected genes (CAV1, COL6A2, FABP4, FBLN1, PCOLCE, and CSPG4) were not associated with overall survival (OS) based on the TCGA BLCA dataset; **(I)** CSPG4 mutation was associated with infiltration levels of CD4^+^ T cells and natural killer (NK) cells.

In addition, GSVA and Pearson correlation analysis identified PCOLCE, CSPG4, COL6A2, and CAV1 were positively correlated with the score of GEM-resistance (R-value >0, p < 0.05) (Figures 9A–D). However, FBLN1 and FABP4 were negatively correlated with GEM-resistance (R-value <0, p < 0.05) (Figures 9E,F).

### Genetic mutations of hub genes in TCGA BLCA dataset

Figure 9G illustrated the mutation frequencies of the six hub genes (CAV1, COL6A2, FABP4, FBLN1, PCOLCE, and CSPG4) in 404 BCa patients from the TCGA BLCA dataset. Among the six hub genes, the top three most frequently mutated genes were FBLN1 (5.0%), FABP4 (4.0%), and CSPG4 (4.0%). FBLN1 mutations included fusion mutation, amplification mutation, and deletion mutation. Most of FABP4 mutations were amplification mutations and most of CSPG4 mutations were missense mutations.

Furthermore, mutations of all the six hub genes didn’t influence the OS time in comparison with the wild-type group (p > 0.05) (Figure 9H). TIMER identified that CSPG4 mutations could elevate the abundance of CD4^+^ T cells (p = 0.009) and NK cells (p = 0.021) (Figure 9I), while the genetic mutations of the other five hub genes were not associated with abundance of immune cells.

### Construction of miRNA-gene regulatory network

**FIGURE 10:**
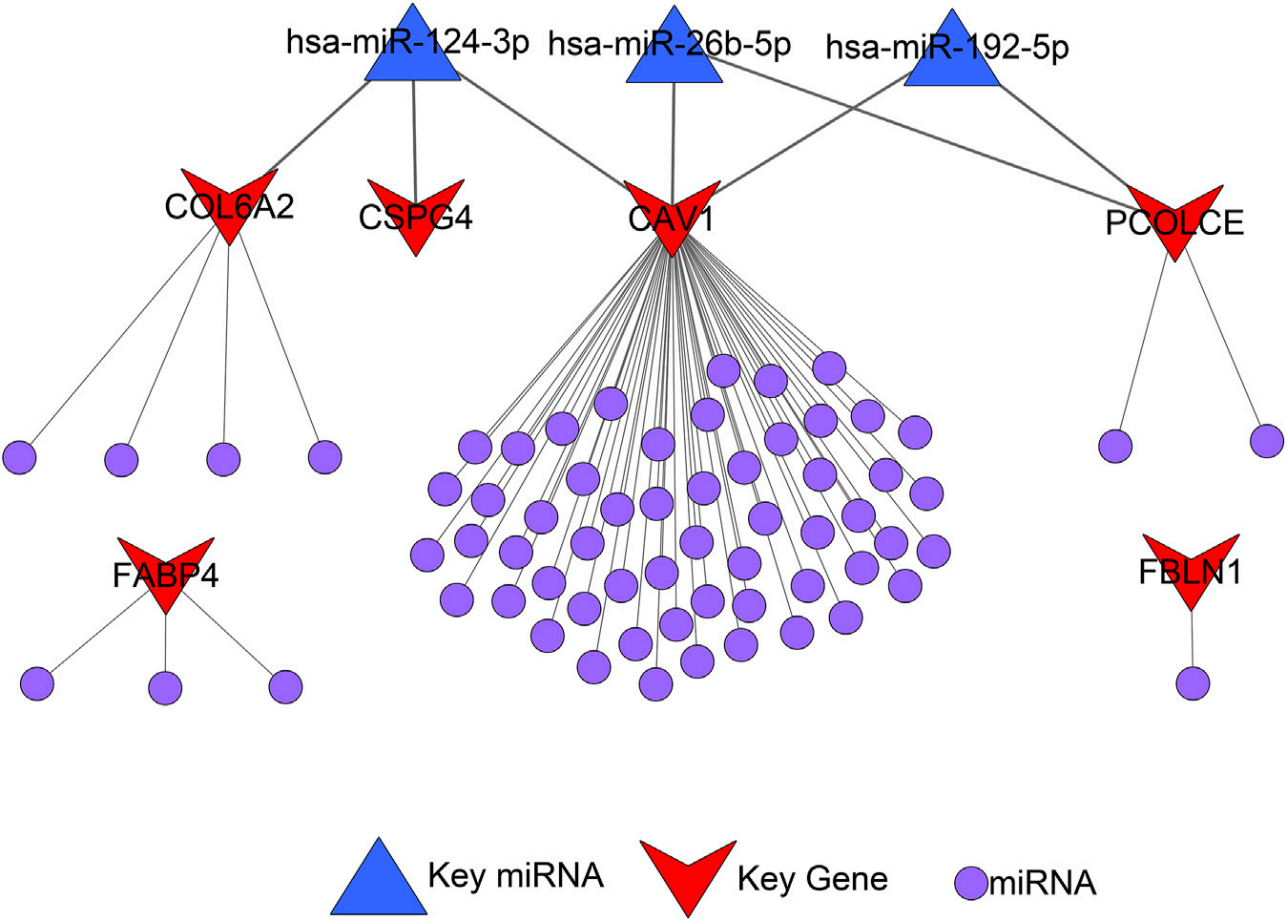
microRNA-gene regulatory network.

A total of 72 miRNAs might regulate the expression levels of six hub genes through the miRTarBase and Targetscan databases (Table 5). The miRNA-gene regulatory network is displayed in Figure 10.

**TABLE 5:** Upstream microRNAs (miRNAs) of the six hub genes (CAV1, COL6A2, FABP4, FBLN1, PCOLCE and CSPG4)

Hub gene	Upstream miRNAs
**CAV1**	**hsa-miR-124-3p**, **hsa-miR-26b-5p**, **hsa-miR-192-5p**, hsa-miR-34c-5p, hsa-miR-34b-5p, hsa-miR-103a-3p, hsa-miR-7-5p, hsa-miR-199a-5p, hsa-miR-203a-3p, hsa-miR-107, hsa-miR-17-5p, hsa-miR-20a-5p, hsa-miR-93-5p, hsa-miR-106a-5p, hsa-miR-194-5p, hsa-miR-106b-5p, hsa-miR-20b-5p, hsa-miR-526b-3p, hsa-miR-519d-3p, hsa-miR-3609, hsa-miR-548ah-5p, hsa-miR-4796-3p, hsa-miR-3973, hsa-miR-873-5p, hsa-miR-520h, hsa-miR-520g-3p, hsa-miR-4463, hsa-miR-1238-3p, hsa-miR-6749-3p, hsa-miR-6792-3p, hsa-miR-4691-5p, hsa-miR-627-3p, hsa-miR-660-3p, hsa-miR-5193, hsa-miR-670-3p, hsa-miR-4277, hsa-miR-584-3p, hsa-miR-5004-3p, hsa-miR-1261, hsa-miR-4791, hsa-miR-3201, hsa-miR-766-5p, hsa-miR-3140-3p, hsa-miR-4722-5p, hsa-miR-4468, hsa-miR-4673, hsa-miR-4645-5p, hsa-miR-4692, hsa-miR-4514, hsa-miR-4459, hsa-miR-556-5p, hsa-miR-208b-5p, hsa-miR-208a-5p, hsa-miR-6165, hsa-miR-6753-5p, hsa-miR-1911-3p, hsa-miR-338-5p, hsa-miR-4517
**COL6A2**	**hsa-miR-124-3p**, hsa-miR-10b-5p, hsa-miR-10a-5p, hsa-miR-3928-3p, hsa-miR-29c-3p
**PCOLCE**	**hsa-miR-192-5p**, **hsa-miR-26b-5p**, hsa-miR-215-5p, hsa-miR-182-5p
**FABP4**	hsa-miR-138-5p, hsa-miR-369-5p, hsa-miR-335-5p
**FBLN1**	hsa-miR-30a-3p
**CSPG4**	**hsa-miR-124-3p**

Bold miRNAs meant key miRNAs regulating ≥2 hub genes.

**FIGURE 11:**
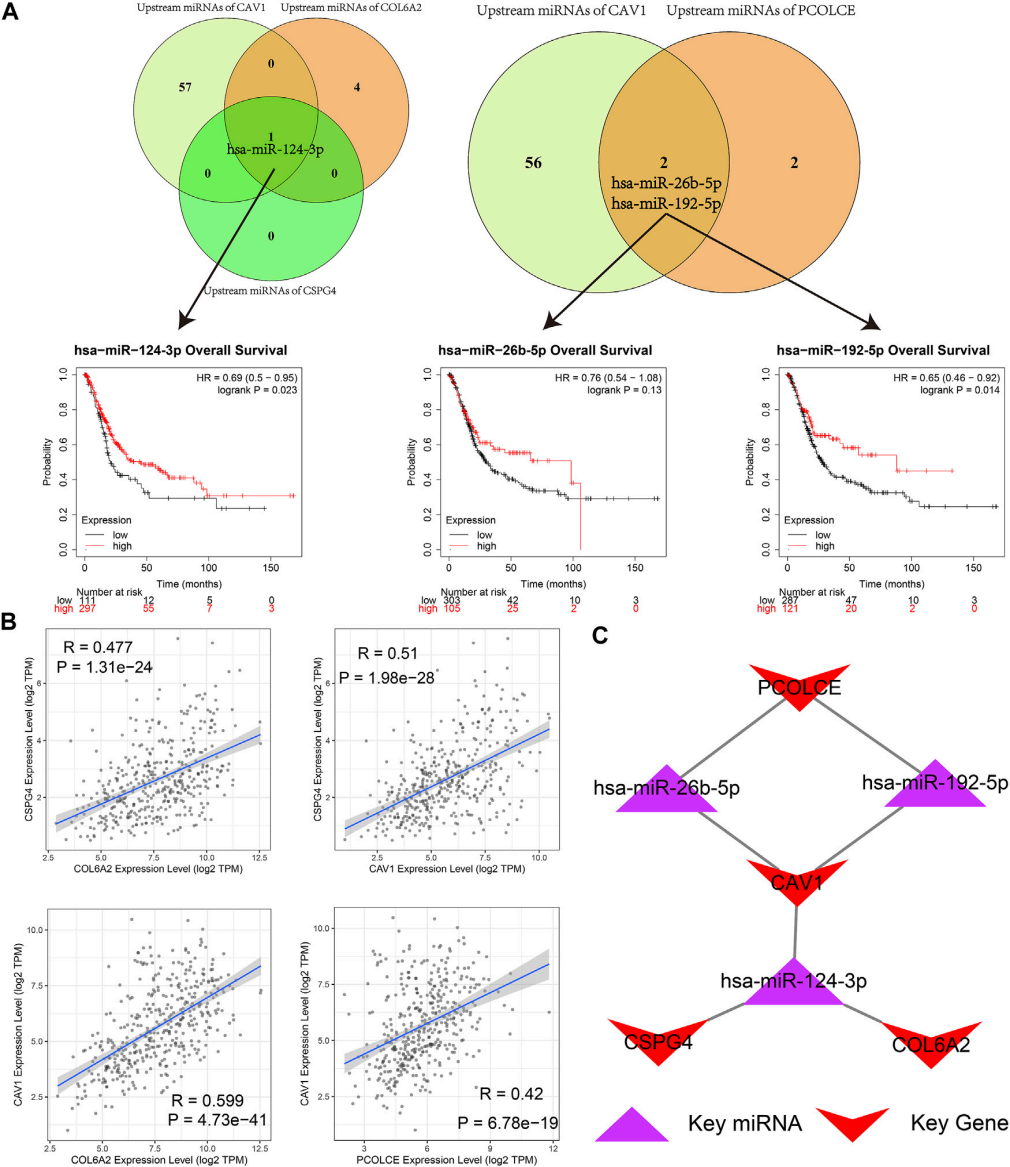
Identification of hub microRNAs (miRNAs) and the key miRNA-gene regulatory network. **(A)** hsa-miR-124-3p, hsa-miR-26b-5p, and hsa-miR-192-5p were overlapped upstream miRNAs of four hub genes (CAV1, COL6A2, PCOLCE, and CSPG4) and their survival analyses; **(B)** Pairwise correlation analyses of four hub genes (CAV1, COL6A2, PCOLCE, and CSPG4); **(C)** Key miRNA-gene regulatory network formed by the three overlapped miRNAs and the four hub genes.

As we could see in Figure 11A, hsa-miR-124-3p was the overlapped upstream miRNA of CAV1, COL6A2, and CSPG4; hsa-miR-26b-5p and hsa-miR-192-5p were overlapped upstream miRNAs of CAV1 and PCOLCE, which indicated the three miRNAs might be key miRNAs in regulating the hub genes. Survival analysis demonstrated that higher expression levels of hsa-miR-124-3p and hsa-miR-192-5p were significantly related to better prognosis in BCa patients from the TCGA BLCA dataset.

Furthermore, a pairwise correlation analysis of CAV1, COL6A2, CSPG4, and PCOLCE was carried out. Expression of the four genes demonstrated a significant positive correlation (p < 0.05). The increase of one hub gene was strongly correlated with the increase of another one (Figure 11B). Hence, based on the overlapped upstream miRNAs and correlation analysis, we confirmed that the three key miRNAs (hsa-miR-124-3p, hsa-miR-26b-5p, and hsa-miR-192-5p) could regulate the four hub genes (CAV1, COL6A2, CSPG4, and PCOLCE) in GEM-resistance and tumor immune microenvironment. We also plotted the key regulatory network in Figure 11C. In addition, we detected DEGs between high score of GEM-resistance and low score of GEM-resistance. We found PCOLCE, CSPG4, COL6A2, and CAV1 were up-regulated in patients with high GEM-resistance scores, which further confirmed their key roles in GEM-resistance (Supplementary Figure S7).

### Gene set enrichment analysis

**FIGURE 12:**
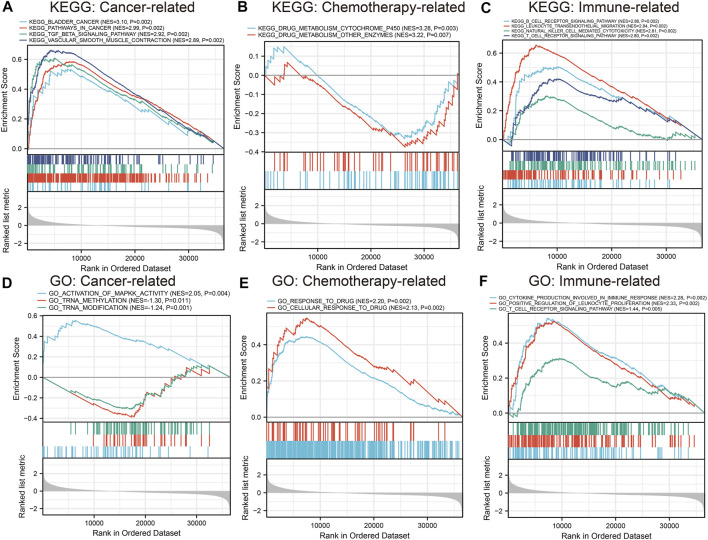
Gene set enrichment analysis of CSPG4. Kyoto Encyclopedia of Genes and Genomes (KEGG) pathway analysis and Gene Ontology (GO) functional analysis identified that CSPG4 was enriched in cancer-related, chemotherapy-related, and immune-related functions. **(A)** Cancer-related KEGG pathway analysis; **(B)** Chemotherapy-related KEGG pathway analysis; **(C)** Immune-related KEGG pathway analysis; **(D)** Cancer-related GO enrichment analysis; **(E)** Chemotherapy-related GO enrichment analysis; **(F)** Immune-related GO enrichment analysis.

GSEA was conducted to investigate the possible role of the six hub genes (CAV1, COL6A2, FABP4, FBLN1, PCOLCE, and CSPG4) involved in GEM-resistance. We identified CSPG4 (Figure 12) was obviously enriched in cancer-related pathways and functions including the bladder cancer pathway (hsa05219) and TGF-β signaling pathway (hsa04350). In addition, CSPG4 exerted a vital role in chemotherapy-related functions including drug metabolism of cytochrome P450 (hsa00982), drug metabolism of other enzymes (hsa00983), cellular response to drug (GO:0035690), and response to drug (GO:0042493). Further, CSPG4 was also enriched in pathways of immune response and immune cells including the B cell receptor signaling pathway (hsa04662), NK cell-mediated cytotoxicity pathway (hsa04650), and T cell receptor signaling pathway (hsa04660) (Supplementary Tables S3, S4). In addition, the other five genes were also enriched in cancer-related (hsa05200 and hsa05219), immune-related (hsa04660, hsa4650, and hsa04662), and chemotherapy-related (hsa00982 and hsa00983) pathways (Supplementary Table S5).

### Selection of targeted drugs for GEM-resistant BCa

CMAP analysis indicated 75 drugs might have antagonistic or synergistic effects on GEM-resistance. According to the enrichment score, Table 6 displayed the top 10 drugs with antagonism and the top 10 drugs with synergism, respectively. The top 10 antagonistic drugs for GEM-resistant BCa were lisinopril, rifabutin, clonidine, prasterone, vorinostat, prednisone, nifenazone, alvespimycin, trichostatin A, and tanespimycin.

**TABLE 6:** Connectivity map (CMAP) database analysis

Type	Rank	CMAP name	Enrichment	p-Value
Antagonistic drugs	1	Lisinopril	−0.898	0.002
	2	Rifabutin	−0.882	0.003
	3	Clonidine	−0.819	0.002
	4	Prasterone	−0.787	0.004
	5	Vorinostat	−0.752	<0.001
	6	Prednisone	−0.685	0.007
	7	Nifenazone	−0.675	0.008
	8	Alvespimycin	−0.532	0.001
	9	Trichostatin A	−0.460	<0.001
	10	Tanespimycin	−0.421	<0.001
Synergistic drugs	1	Oxybuprocaine	0.874	<0.001
	2	Ascorbic acid	0.870	<0.001
	3	Disopyramide	0.790	0.004
	4	Benzocaine	0.788	0.004
	5	Eticlopride	0.782	0.004
	6	Sisomicin	0.770	0.005
	7	Viomycin	0.748	0.008
	8	Midodrine	0.726	0.004
	9	6-Bromoindirubin-3′-oxime	0.677	0.001
	10	Fludrocortisone	0.541	0.010

## Discussion

Accumulating evidence indicates that aberrantly expressed genes are significantly associated with GEM-resistance in BCa. It is reported that CSNK1D played a key role in the metabolism of GEM, and inhibition of CSNK1D could sensitize BCa cells to GEM treatment, which might be utilized as a therapeutic target for metastatic BCa (Udhaya Kumar et al., 2020; Vena et al., 2020). Xie et al. identified that circular RNA (circRNA) circHIPK3 was an independent prognostic predictor, and up-regulation of circHIPK3 promoted GEM-sensitivity in BCa (Xie et al., 2020). Another study based on BCa cells indicated that inhibition of GP130 could enhance the sensitivity to GEM and reduce viability and migration of tumor cells through regulating the PI3K/AKT/mTOR signaling pathways (Li X. et al., 2019). This study analyzed the GSE77883 dataset from the GEO and TCGA BLCA datasets to identify promising biomarkers for GEM-resistant BCa through high-throughput sequencing data and bioinformatics analyses.

We assessed immune cells and identified that both BCa development and GEM-resistance were immune-related. We found that 82 key DEGs were significantly related to both BCa development and GEM-resistance. Functional enrichment analyses found these key DEGs were enriched in immune-related items, especially in the regulation of immune cell proliferation. After construction of the PPI network and Cox regression analysis, we selected six hub genes (CAV1, COL6A2, FABP4, FBLN1, PCOLCE, and CSPG4) with the highest connectivity degrees and prognostic values for further analyses. We used immunohistochemistry from THPA and expression profiles from larger samples to confirm the down-regulation of six hub genes in BCa at protein and mRNA level, respectively. Survival analyses demonstrated that they were related to OS time. Down-regulation of CSPG4, CAV1, and PCOLCE might be related to elevated chemotherapy sensitivity and thus lower expression levels of them were associated with better OS. IMvigor210 cohort validated that COL6A2, FABP4, and FBLN1 could predict the OS after immunotherapy with atezolizumab. Pearson correlation analysis revealed CAV1, COL6A2, and PCOLCE had strong correlations with immune cells, such as dendritic cells and macrophages. Next, we constructed the key miRNA-gene regulatory network based on four key genes (CAV1, COL6A2, PCOLCE, and CSPG4) and three key miRNAs (hsa-miR-124-3p, hsa-miR-26b-5p, and hsa-miR-192-5p).

CAV1 is the chief component of the caveolae plasma membranes in most human cells and participates in immune response and cancer progression (Shi et al., 2020). CAV1 in prostate cancer could induce epithelial–mesenchymal transition through activating cancer immune evasion, and CAV1 in cancer-derived exosomes was able to induce chemoresistance in recipient cells (Lin et al., 2019), which conformed to our results revealed in GEM-resistant BCa. In addition, CAV1 might also promote systemic lupus erythematosus through regulating pathways of T cell costimulation, lymphocyte costimulation, and B cell receptor signaling (Udhaya Kumar et al., 2020). CAV1 was pivotal in acute immune-mediated hepatic damage through driving RNS-mediated ferroptosis (Deng et al., 2020). The presently found CAV1 was the key gene regulated by three upstream miRNAs in the miRNA-gene regulatory network. Zhou et al. found that hsa-miR-124-3p and hsa-miR-192-5p suppressed the proliferation and invasion of tumor cells by targeting CAV1 (Zhou et al., 2018; Chen et al., 2019). Therefore, we hypothesized that CAV1 could facilitate tumor development and GEM-resistance via immune escape mechanism.

COL6A2 is one of the collagen family members and encodes one alpha chain of type VI collagen identified in most connective tissues (Hou et al., 2016). COL6A2 was reported to be up-regulated and to gather in the ECM-receptor interaction signaling pathway, which promoted the BCa progression (Zhu et al., 2019). Down-regulation of COL6A2 induced by decreased IDO1 could suppress host anti-tumor immune response through inhibiting immune-related pathways (Xiang et al., 2019). We found COL6A2 was positively correlated with most immune cells in a tumor-immune microenvironment, which could support the highly immunogenic nature of COL6A2 in BCa.

CSPG4 is a kind of transmembrane proteoglycan, considered as a promising tumor-associated antigen (Cavallo et al., 2007). Previous investigations have identified CSPG4 as a key gene in soft-tissue sarcoma, melanoma, and glioblastoma (Benassi et al., 2009; Wang et al., 2011). We found CSPG4 exerted a vital role in BCa prognosis, and both the expression and mutation of CSPG4 might influence the immune cells in BCa. In addition, Rolih et al. systematically summarized the evidence of CSPG4 in tumor biology and suggested that CSPG4 and anti-CSPG4 vaccination strategy had the potential to be an attractive target for anti-tumor immunotherapy (Rolih et al., 2017). GSEA detected that CSPG4 contributed to cancer-related pathways, immune system process, and drug metabolism, which further confirmed its value in drug-resistance and immunotherapy of BCa (Song et al., 2020). Nevertheless, further investigations are demanded to verify its mechanisms in BCa.

PCOLCE is a glycoprotein that elevates the activity of procollagen C-proteinase (Pulido et al., 2018). PCOLCE was up-regulated in osteosarcoma and promotes the distant metastasis (Wang et al., 2019). We found PCOLCE was down-regulated and was also associated with prognosis in BCa. The difference of PCOLCE expression between the two tumors could be explained by the miRNA-gene regulatory network; hsa-miR-26b-5p and hsa-miR-192-5p regulated the co-expression of PCOLCE and CAV1 in the network of BCa. Besides, PCOLCE was revealed to be involved in platelet and endothelial function and immune activation in human immunodeficiency virus (HIV) patients after pitavastatin treatment, which bound PCOLCE to immune related functions (DeFilippi et al., 2020).

The identified key miRNA-gene regulatory network indicated that hsa-miR-124-3p, hsa-miR-192-5p, and hsa-miR-26b-5p were key miRNAs in regulating the above genes. It is reported that higher expression level of hsa-miR-124-3p suppressed tumor proliferation and indicated better BCa prognosis in BCa through targeting downstream genes (Wang et al., 2015; Zhou et al., 2018; Zo and Long, 2019). Besides, hsa-miR-124-3p was frequently and tumor-specifically methylated in primary BCa, indicating that epigenetic silencing of hsa-miR-124-3p may also participate in BCa development (Shimizu et al., 2013). *In vitro* studies demonstrated that hsa-miR-192-5p was a suppressor for BCa cells by cell cycle regulation and clinical studies identified hsa-miR-192-5p as an independent prognostic marker based on multivariate COX regression (Jin et al., 2015; Hu et al., 2017). Bioinformatics analysis combined with *in vitro* experiments demonstrated that hsa-miR-26b-5p was a critical regulator in BCa progression by targeting the proliferation-related gene PDCD10 (Wu et al., 2018). With regard to the relationship between the three miRNAs and immune system, there is evidence that they played roles in modulating immune escape and immune cells (Li K. et al., 2019; Ji et al., 2019; Amoruso et al., 2020).

The CMAP database analysis identified the potential drugs with synergism or antagonism to GEM-resistance. We identified that two histone deacetylase inhibitors (HDACIs), trichostatin A and vorinostat, had antagonistic effects on GEM-resistance, indicating that the two drugs might circumvent the GEM-resistance and enhance the sensitivity to GEM. *In vivo* studies based on BCa cells revealed that trichostatin A may synergistically enhance GEM-mediated cell cycle arrest and apoptosis through inhibiting the Raf/MEK/ERK pathway (Jeon et al., 2011; Lin et al., 2018), which provided HDACIs as promising treatment methods to improve GEM-resistant BCa patients in future clinical practice. It is also worth noticing that oxybuprocaine and benzocaine had the synergistic effects to GEM-resistance and the two anesthetics might aggravate GEM-resistance in BCa. Furthermore, ascorbic acid, also called vitamin C, was identified to be associated with aggravate GEM-resistance. However, a phase I clinical trial based on pancreatic cancer patients indicated that ascorbic acid combined concurrently with GEM was well tolerated and could reduce adverse events, which is inconsistent with our results (Welsh et al., 2013).

In order to reduce the potential bias caused by one single method and to verify the relationship between key biomarkers in GEM-resistant BCa and tumor immune microenvironment, the present study used two methods including MCPcounter (Becht et al., 2016) and TIMER (Li et al., 2016; Li et al., 2017) for the calculating of the immune cells. By using these two methods separately, the results indicated that GEM-resistance and the key genes were closely related to immune cells, which further verified that the results were stable and convincing. However, several limitations existed. Even if immunohistochemistry was used to confirm the down-regulation of hub genes, and cell lines instead of *in vivo* models were used to perform CMAP analysis, we didn’t verify their actual molecular mechanisms. Therefore, the above hypotheses should be verified through experimental methods in future studies. Since most BCa tissues from the TCGA BLCA dataset were MIBC tissues, our findings were more applicable to GEM-resistant MIBC patients.

## Conclusion

We identified both BCa development and GEM-resistance were immune-related. CAV1, COL6A2, FABP4, FBLN1, PCOLCE, and CSPG4 are hub genes in GEM-resistant MIBC. They could serve as potential prognostic predictors and immunotherapy targets for MIBC. In addition, the key miRNA-gene regulatory network suggested three key miRNAs (hsa-miR-124-3p, hsa-miR-26b-5p, and hsa-miR-192-5p) might also be implicated in GEM-resistance. Ultimately, CMAP analysis identified HDACIs (trichostatin A and vorinostat) might circumvent the GEM-resistance and enhance the sensitivity to GEM.

## Data Availability

The datasets presented in this study can be found in online repositories. The names of the repository/repositories and accession number(s) can be found in the article/Supplementary Material.
