# Supplementary material for: Gemcitabine-Resistant Biomarkers in Bladder Cancer are Associated with Tumor-Immune Microenvironment
Source: Front Cell Dev Biol. 2022 Jan 21;9:809620. doi: 10.3389/fcell.2021.809620 (PMC8814447; doi:10.3389/fcell.2021.809620)
Supplement: Supplementary file 1 [file DataSheet1.doc]

Supplementary Material

**Supplementary Figures**

**
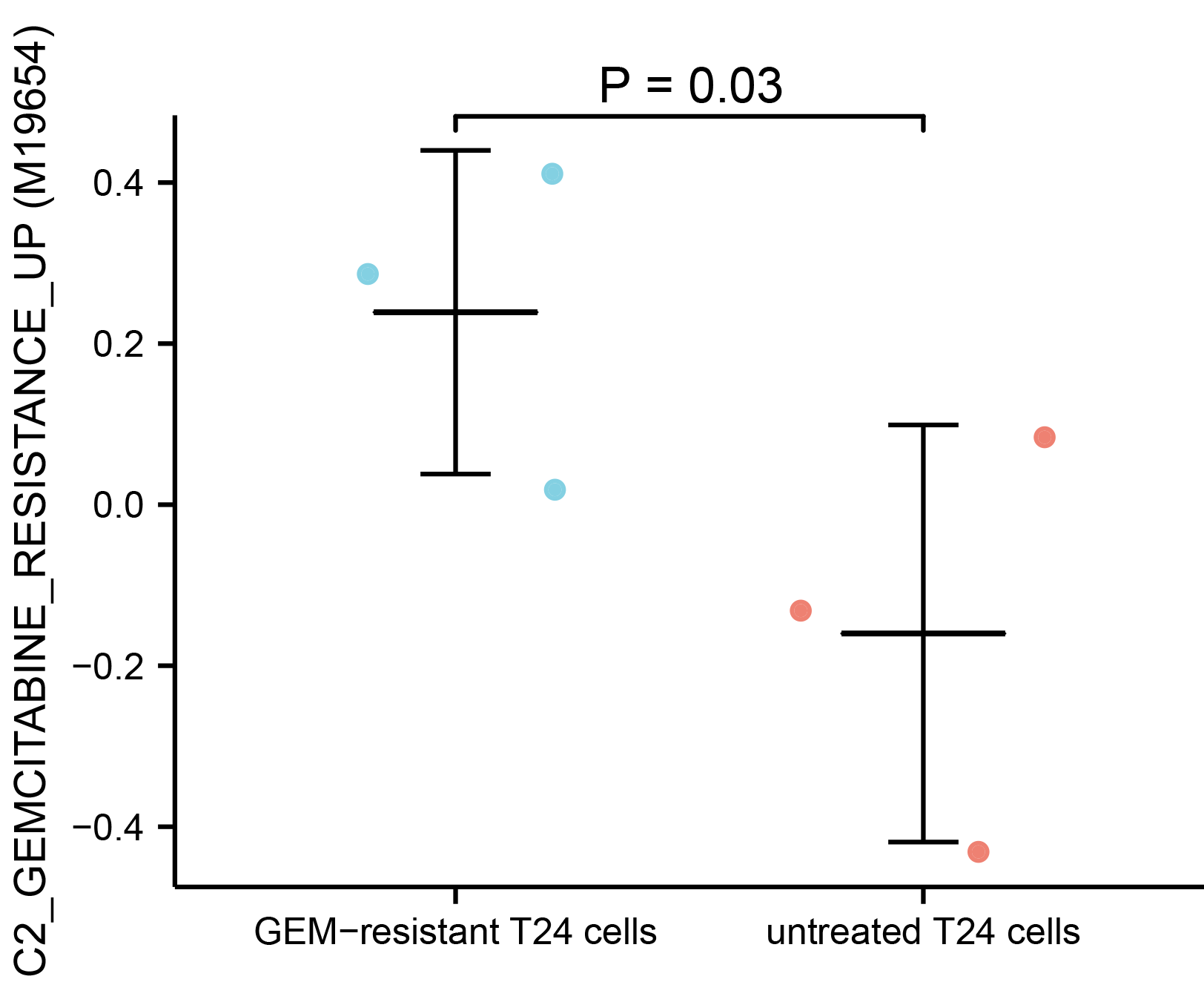
**

**Supplementary Figure 1.** Gene set variation analysis identified the score of gemcitabine(GEM)-resistance was higher in GEM-resistant T24 cells than untreated T24 cells from GSE77883 dataset.

**
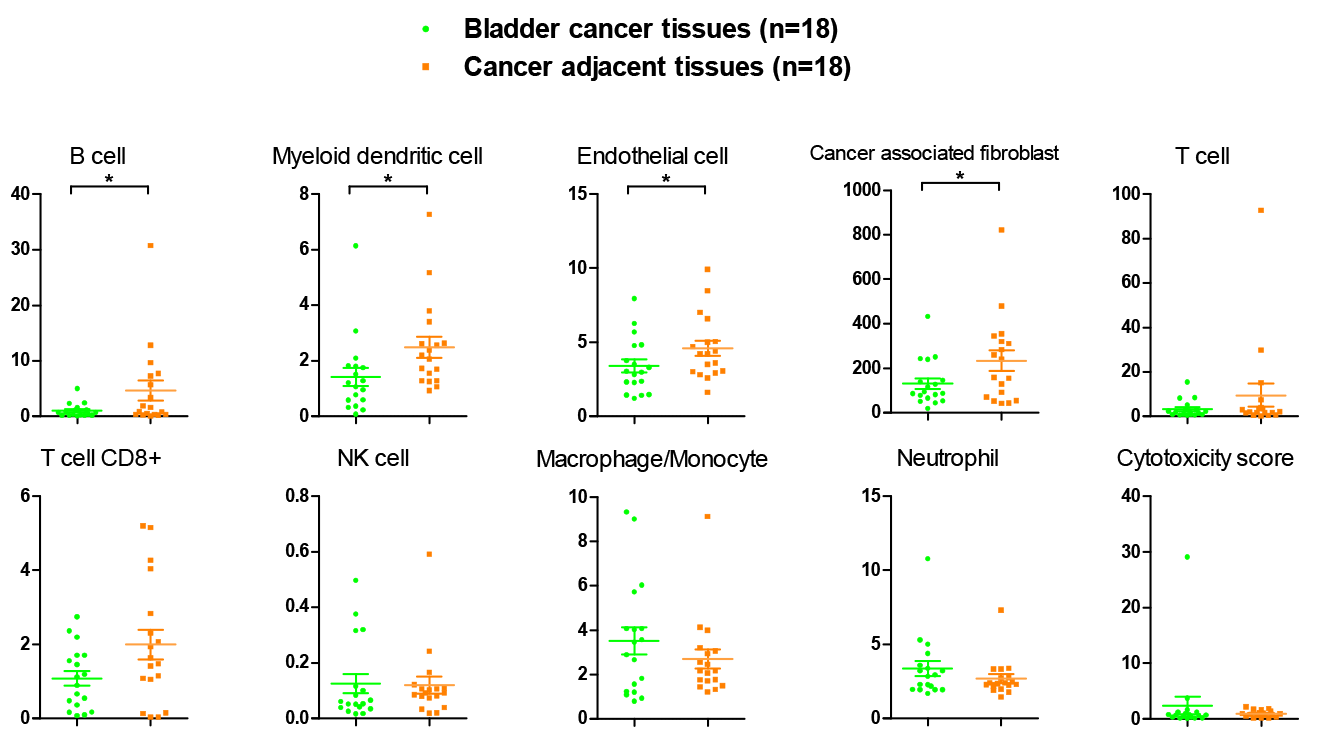
**

**Supplementary Figure 2.** Tumor immune microenvironment in bladder cancer (BCa) development based on 18 pairs of BCa tissues and matched adjacent normal tissues from TCGA (The Cancer Genome Atlas) BLCA (Bladder Urothelial Carcinoma) dataset. Tumor-infiltrating immune cells analysis between BCa tissues and matched adjacent normal tissues. *** *P*<0.05.**

**
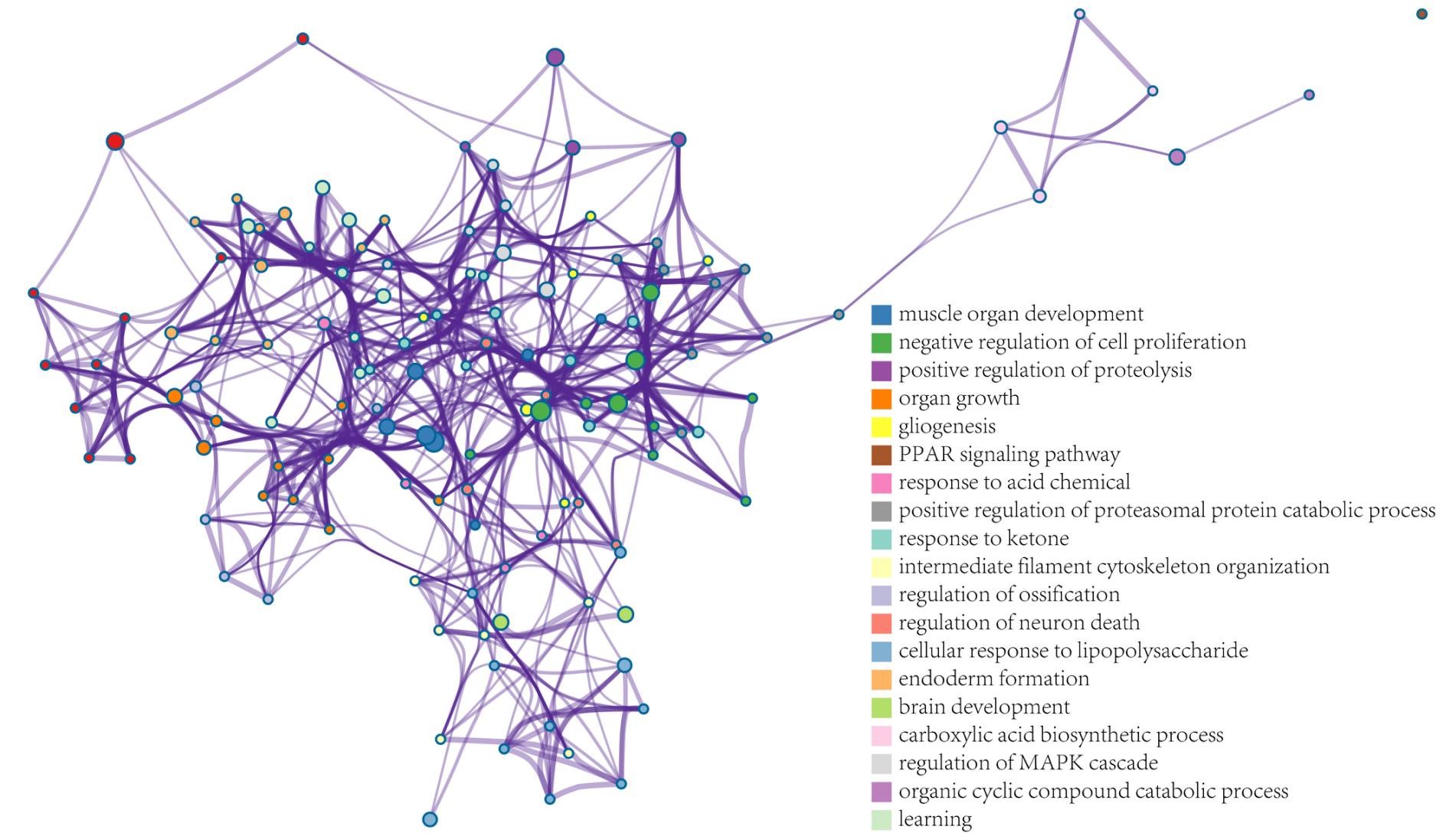
**

**Supplementary Figure 3.** Interactions of the main 19 clustered functional enrichment terms.

**
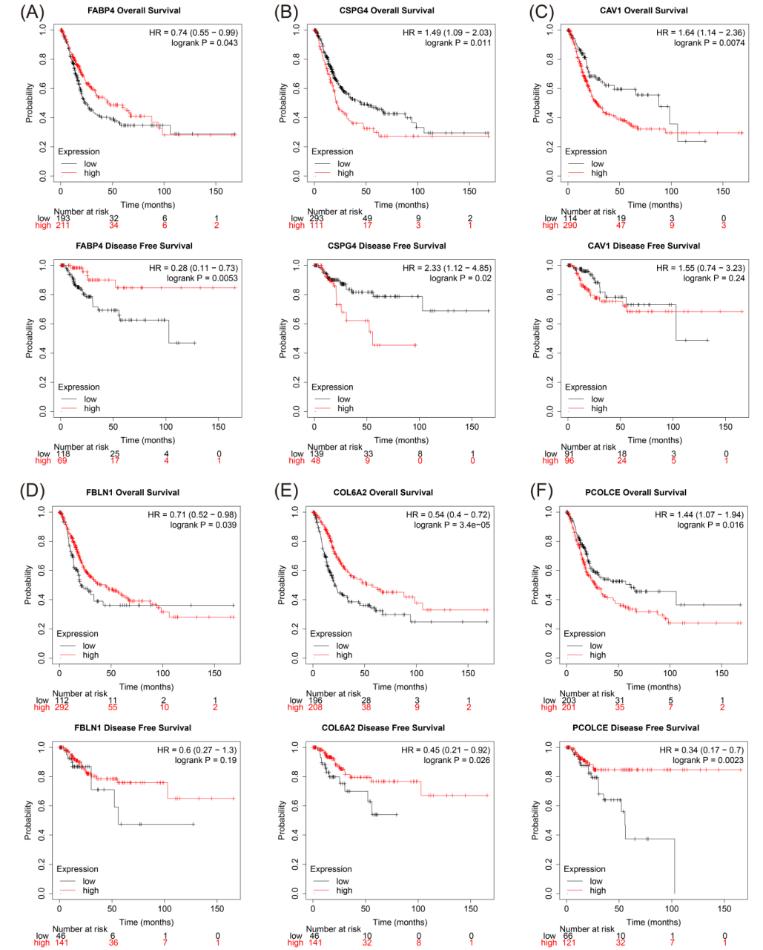
**

**Supplementary Figure 4.** Kaplan-Meier (KM) survival curves showed that the 6 hub genes (CAV1, COL6A2, FABP4, FBLN1, PCOLCE and CSPG4) were associated with overall survival (OS) and disease-free survival (DFS) based on TCGA BLCA dataset.

**(A)** FABP4; **(B)** CSPG4; **(C)** CAV1; **(D)** FBLN1; **(E)** COL6A2; **(F)** PCOLCE.

**
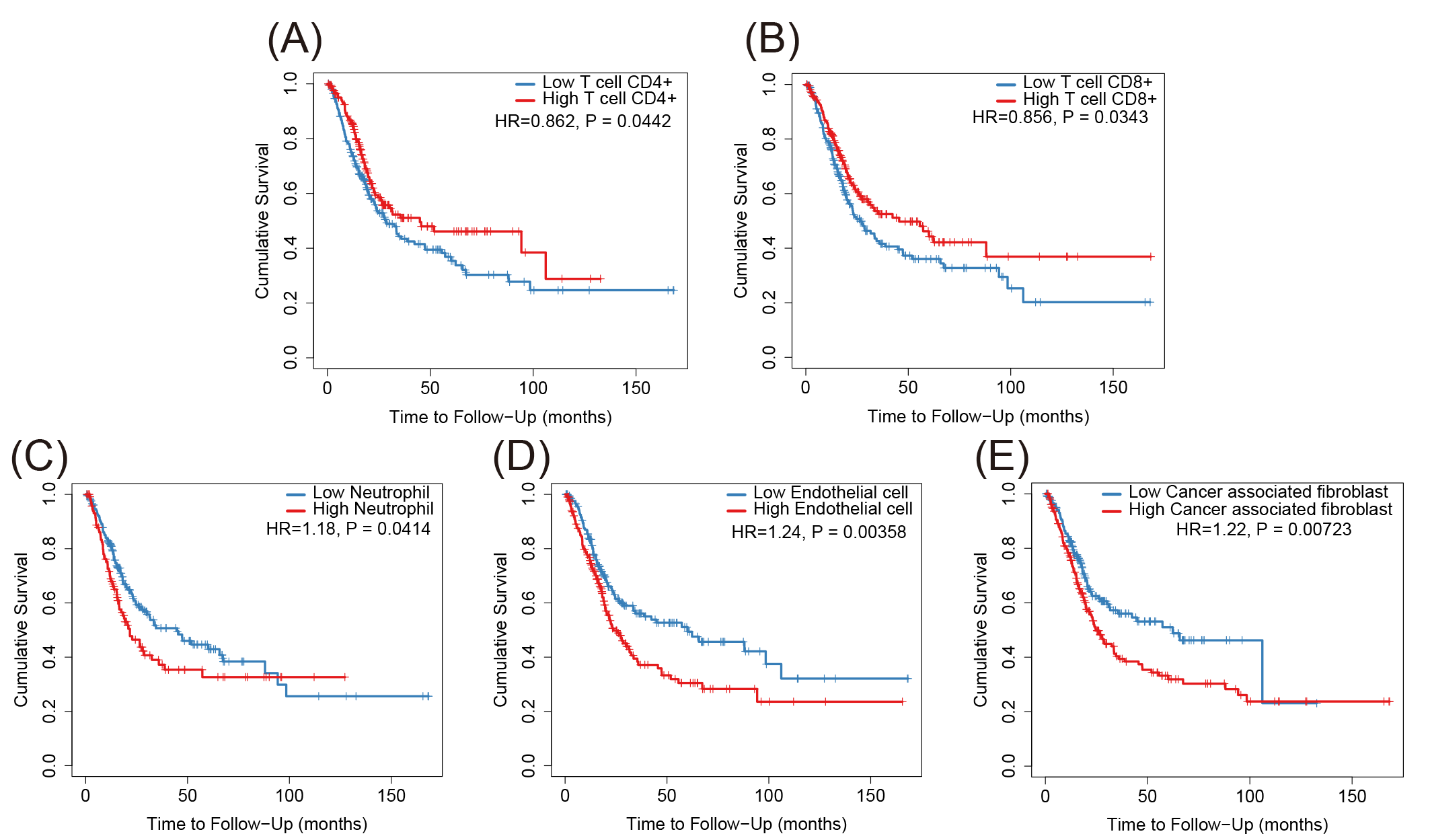
**

**Supplementary Figure 5.** Kaplan-Meier (KM) survival curves showed that tumor-infiltrating immune cells were associated with overall survival (OS) based on TCGA BLCA dataset.

**(A)** CD4+ T cells; **(B)** CD8+ T cells; **(C)** Neutrophils; **(D)** Endothelial cells; **(E)** Cancer associated fibroblast cells.

**
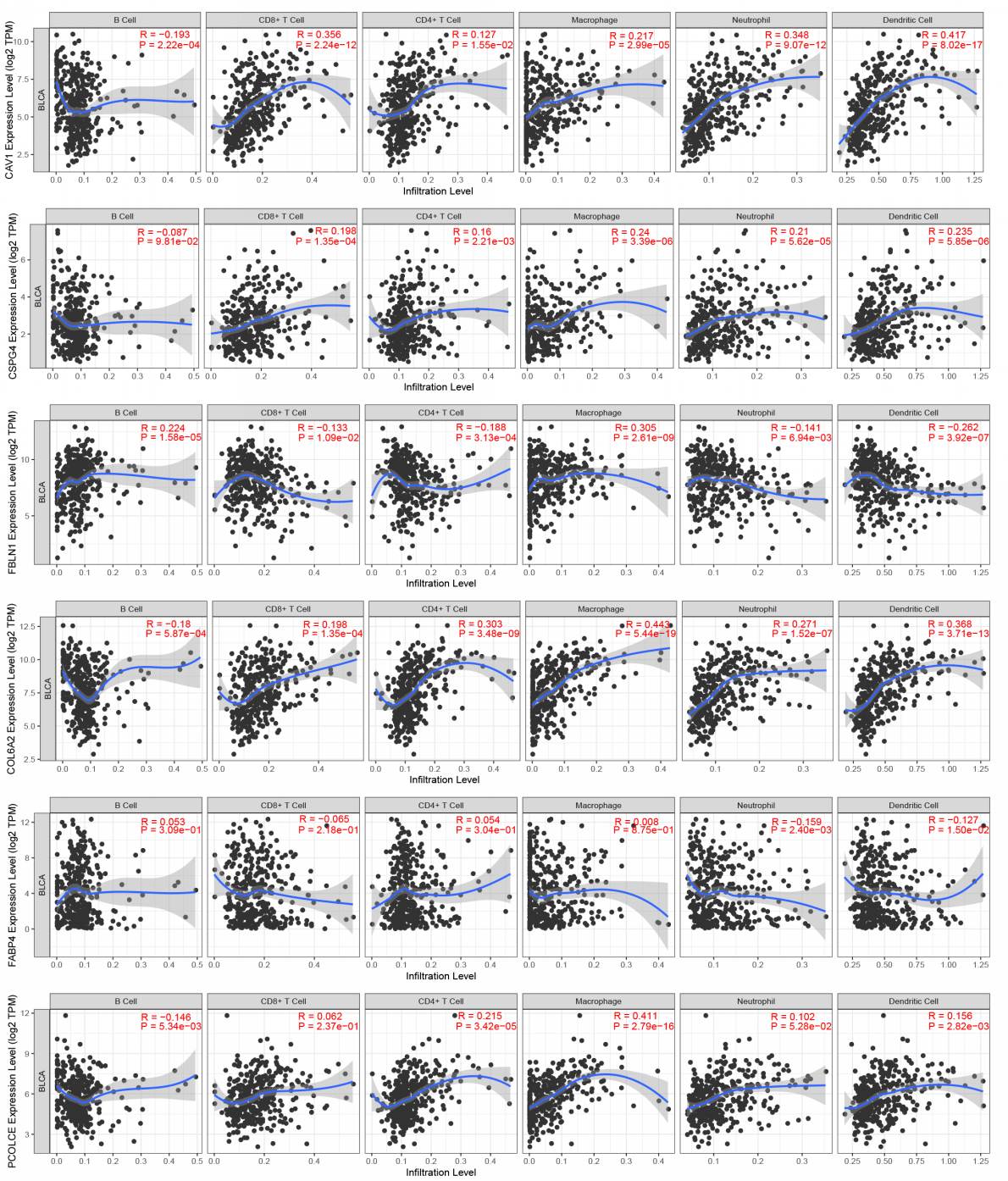
Supplementary Figure 6.** Correlation analysis of immune cells and the 6 hub genes (CAV1, COL6A2, FABP4, FBLN1, PCOLCE and CSPG4).

**
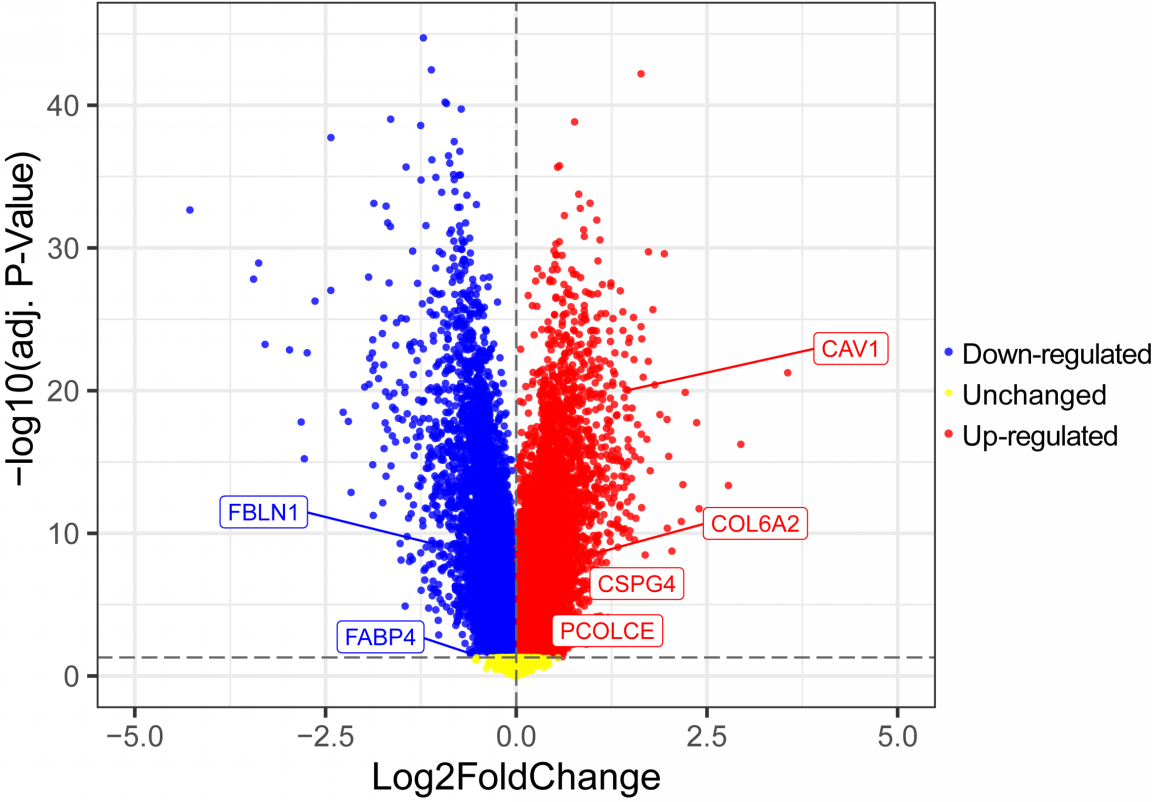
Supplementary Figure 7.** Volcano plot of DEGs between high GEM-resistance score group and low GEM-resistance score group based on TCGA BLCA dataset. CAV1, COL6A2, PCOLCE and CSPG4 were up-regulated in high GEM-resistance score group.

**Supplementary Tables**

**Supplementary Table 1.** Summary of overlapped differentially expressed genes (DEGs) in both gemcitabine-resistance and bladder cancer (BCa) development.

| DEGs | logFC in GSE77883 | logFC in TCGA | Type |
| --- | --- | --- | --- |
| HS6ST2 | 6.77 | 1.01 | Up-regulation |
| MAGEA6 | 3.24 | 1.65 | Up-regulation |
| CST1 | 3.02 | 2.14 | Up-regulation |
| KLC3 | 2.84 | 1.04 | Up-regulation |
| LYPD6B | 2.61 | 1.15 | Up-regulation |
| EPHA4 | 1.78 | 1.05 | Up-regulation |
| LPAR2 | 1.63 | 1.22 | Up-regulation |
| STEAP3 | 1.24 | 1.21 | Up-regulation |
| OCIAD2 | 1.20 | 1.00 | Up-regulation |
| PSAT1 | 1.12 | 1.18 | Up-regulation |
| SULT1E1 | 1.05 | 1.23 | Up-regulation |
| EFEMP1 | -1.03 | -2.25 | Down-regulation |
| EPB41L2 | -1.07 | -1.08 | Down-regulation |
| SRGN | -1.12 | -1.14 | Down-regulation |
| PDLIM4 | -1.12 | -1.82 | Down-regulation |
| TACC2 | -1.15 | -1.46 | Down-regulation |
| FAM43A | -1.16 | -1.57 | Down-regulation |
| WLS | -1.18 | -1.26 | Down-regulation |
| NNAT | -1.19 | -1.91 | Down-regulation |
| OAT | -1.21 | -1.21 | Down-regulation |
| IL6ST | -1.27 | -1.58 | Down-regulation |
| CAP2 | -1.30 | -1.34 | Down-regulation |
| APOLD1 | -1.31 | -1.85 | Down-regulation |
| SOX7 | -1.33 | -1.08 | Down-regulation |
| FERMT2 | -1.34 | -2.03 | Down-regulation |
| ASS1 | -1.36 | -1.32 | Down-regulation |
| SH3BGR | -1.41 | -1.64 | Down-regulation |
| SYNM | -1.43 | -4.35 | Down-regulation |
| CSPG4 | -1.43 | -1.52 | Down-regulation |
| JDP2 | -1.44 | -1.39 | Down-regulation |
| IGFBP6 | -1.44 | -2.34 | Down-regulation |
| NFIA | -1.46 | -1.30 | Down-regulation |
| PNMA1 | -1.48 | -1.12 | Down-regulation |
| TPPP | -1.51 | -2.29 | Down-regulation |
| VIM | -1.53 | -1.43 | Down-regulation |
| MAP3K8 | -1.54 | -1.44 | Down-regulation |
| SCARA3 | -1.55 | -1.46 | Down-regulation |
| BTG2 | -1.56 | -1.31 | Down-regulation |
| RBP4 | -1.63 | -1.00 | Down-regulation |
| COL6A1 | -1.63 | -1.17 | Down-regulation |
| PLIN4 | -1.65 | -2.84 | Down-regulation |
| PRICKLE2 | -1.67 | -1.59 | Down-regulation |
| THBS1 | -1.68 | -1.11 | Down-regulation |
| DPYSL2 | -1.76 | -1.25 | Down-regulation |
| PLCD3 | -1.77 | -1.48 | Down-regulation |
| RASGRP2 | -1.79 | -1.46 | Down-regulation |
| COL6A2 | -1.83 | -1.60 | Down-regulation |
| PCOLCE | -1.84 | -1.02 | Down-regulation |
| PLSCR4 | -1.84 | -1.31 | Down-regulation |
| PDE4B | -1.86 | -1.20 | Down-regulation |
| KCTD12 | -1.93 | -1.27 | Down-regulation |
| HSPA2 | -1.94 | -1.80 | Down-regulation |
| AK4 | -1.95 | -1.04 | Down-regulation |
| PCDH7 | -1.96 | -1.33 | Down-regulation |
| NES | -1.96 | -1.60 | Down-regulation |
| PDE7B | -2.03 | -1.13 | Down-regulation |
| S1PR1 | -2.12 | -1.46 | Down-regulation |
| BCHE | -2.13 | -1.75 | Down-regulation |
| NUPR1 | -2.18 | -1.29 | Down-regulation |
| CAV1 | -2.18 | -2.15 | Down-regulation |
| EPHX2 | -2.28 | -1.54 | Down-regulation |
| MGP | -2.29 | -2.75 | Down-regulation |
| PTX3 | -2.35 | -1.62 | Down-regulation |
| COL4A6 | -2.45 | -1.76 | Down-regulation |
| SVIL | -2.60 | -1.70 | Down-regulation |
| ASB2 | -2.63 | -2.43 | Down-regulation |
| FBLN1 | -2.67 | -1.72 | Down-regulation |
| SYNPO | -2.70 | -1.83 | Down-regulation |
| PALM | -2.77 | -1.22 | Down-regulation |
| CYP27A1 | -2.78 | -1.55 | Down-regulation |
| ANKRD35 | -2.82 | -1.20 | Down-regulation |
| IL33 | -3.00 | -2.46 | Down-regulation |
| DKK1 | -3.08 | -2.28 | Down-regulation |
| TGFBR3 | -3.23 | -2.00 | Down-regulation |
| ECSCR | -3.26 | -1.40 | Down-regulation |
| CCL2 | -3.55 | -2.19 | Down-regulation |
| ANGPTL4 | -3.97 | -1.45 | Down-regulation |
| SERPINF1 | -3.98 | -1.85 | Down-regulation |
| CNRIP1 | -4.66 | -1.46 | Down-regulation |
| FABP4 | -4.72 | -2.64 | Down-regulation |
| CFH | -4.90 | -1.57 | Down-regulation |
| SGCE | -10.43 | -1.42 | Down-regulation |

**Supplementary Table 2.** Metascape identified the interactions of the main 19 clustered enrichment terms based on 82 overlapped differentially expressed genes.

| Category | Term | Description | LogP-value | InTerm_InList |
| --- | --- | --- | --- | --- |
| Canonical Pathways | M5884 | NABA CORE MATRISOME | -7.49 | 10/275 |
| Canonical Pathways | M18 | PID INTEGRIN1 PATHWAY | -5.52 | 5/66 |
| GO Biological Processes | GO:0003414 | chondrocyte morphogenesis involved in endochondral bone morphogenesis | -4.61 | 3/17 |
| GO Biological Processes | GO:0003429 | growth plate cartilage chondrocyte morphogenesis | -4.61 | 3/17 |
| GO Biological Processes | GO:0090171 | chondrocyte morphogenesis | -4.61 | 3/17 |
| GO Biological Processes | GO:0003422 | growth plate cartilage morphogenesis | -4.53 | 3/18 |
| Canonical Pathways | M3008 | NABA ECM GLYCOPROTEINS | -4.26 | 6/196 |
| Reactome Gene Sets | R-HSA-2022090 | Assembly of collagen fibrils and other multimeric structures | -4.26 | 4/61 |
| GO Biological Processes | GO:0003418 | growth plate cartilage chondrocyte differentiation | -4.20 | 3/23 |
| GO Biological Processes | GO:0003433 | chondrocyte development involved in endochondral bone morphogenesis | -4.20 | 3/23 |
| Reactome Gene Sets | R-HSA-1474244 | Extracellular matrix organization | -4.13 | 7/301 |
| Reactome Gene Sets | R-HSA-1650814 | Collagen biosynthesis and modifying enzymes | -4.10 | 4/67 |
| GO Biological Processes | GO:0060536 | cartilage morphogenesis | -3.93 | 3/28 |
| GO Biological Processes | GO:0003413 | chondrocyte differentiation involved in endochondral bone morphogenesis | -3.89 | 3/29 |
| KEGG Pathway | hsa04512 | ECM-receptor interaction | -3.75 | 4/82 |
| GO Biological Processes | GO:0003417 | growth plate cartilage development | -3.72 | 3/33 |
| Reactome Gene Sets | R-HSA-216083 | Integrin cell surface interactions | -3.69 | 4/85 |
| Reactome Gene Sets | R-HSA-1474290 | Collagen formation | -3.60 | 4/90 |
| GO Biological Processes | GO:0030198 | extracellular matrix organization | -3.56 | 7/373 |
| Canonical Pathways | M5887 | NABA BASEMENT MEMBRANES | -3.47 | 3/40 |
| GO Biological Processes | GO:0003416 | endochondral bone growth | -3.44 | 3/41 |
| GO Biological Processes | GO:0060351 | cartilage development involved in endochondral bone morphogenesis | -3.35 | 3/44 |
| Canonical Pathways | M3005 | NABA COLLAGENS | -3.35 | 3/44 |
| Reactome Gene Sets | R-HSA-8948216 | Collagen chain trimerization | -3.35 | 3/44 |
| GO Biological Processes | GO:0098868 | bone growth | -3.32 | 3/45 |
| Canonical Pathways | M198 | PID SYNDECAN 1 PATHWAY | -3.29 | 3/46 |
| GO Biological Processes | GO:0002063 | chondrocyte development | -3.23 | 3/48 |
| KEGG Pathway | hsa04510 | Focal adhesion | -3.23 | 5/199 |
| GO Biological Processes | GO:0043062 | extracellular structure organization | -3.19 | 7/429 |
| GO Biological Processes | GO:0051216 | cartilage development | -3.11 | 5/212 |
| GO Biological Processes | GO:0002062 | chondrocyte differentiation | -3.07 | 4/124 |
| Reactome Gene Sets | R-HSA-186797 | Signaling by PDGF | -2.99 | 3/58 |
| Reactome Gene Sets | R-HSA-1442490 | Collagen degradation | -2.87 | 3/64 |
| GO Biological Processes | GO:0060350 | endochondral bone morphogenesis | -2.76 | 3/70 |
| Canonical Pathways | M160 | PID AVB3 INTEGRIN PATHWAY | -2.67 | 3/75 |
| Reactome Gene Sets | R-HSA-3000178 | ECM proteoglycans | -2.65 | 3/76 |
| GO Biological Processes | GO:0061448 | connective tissue development | -2.59 | 5/278 |
| KEGG Pathway | hsa04974 | Protein digestion and absorption | -2.45 | 3/90 |
| KEGG Pathway | hsa04151 | PI3K-Akt signaling pathway | -2.21 | 5/342 |
| GO Biological Processes | GO:0060349 | bone morphogenesis | -2.20 | 3/110 |
| GO Biological Processes | GO:0048705 | skeletal system morphogenesis | -2.04 | 4/240 |
| GO Biological Processes | GO:0001501 | skeletal system development | -2.03 | 6/529 |
| GO Biological Processes | GO:0007517 | muscle organ development | -6.90 | 11/406 |
| GO Biological Processes | GO:0061061 | muscle structure development | -5.54 | 12/676 |
| GO Biological Processes | GO:0014706 | striated muscle tissue development | -5.15 | 9/390 |
| GO Biological Processes | GO:0060537 | muscle tissue development | -4.99 | 9/409 |
| GO Biological Processes | GO:0060538 | skeletal muscle organ development | -4.65 | 6/167 |
| GO Biological Processes | GO:0007519 | skeletal muscle tissue development | -3.69 | 5/158 |
| GO Biological Processes | GO:0035914 | skeletal muscle cell differentiation | -2.77 | 3/69 |
| GO Biological Processes | GO:0008285 | negative regulation of cell proliferation | -4.95 | 12/773 |
| GO Biological Processes | GO:1904035 | regulation of epithelial cell apoptotic process | -4.79 | 5/93 |
| GO Biological Processes | GO:0048514 | blood vessel morphogenesis | -4.67 | 11/690 |
| GO Biological Processes | GO:0050680 | negative regulation of epithelial cell proliferation | -4.59 | 6/171 |
| GO Biological Processes | GO:0001525 | angiogenesis | -4.49 | 10/594 |
| GO Biological Processes | GO:1904019 | epithelial cell apoptotic process | -4.34 | 5/115 |
| GO Biological Processes | GO:2000351 | regulation of endothelial cell apoptotic process | -4.23 | 4/62 |
| GO Biological Processes | GO:0001568 | blood vessel development | -4.21 | 11/777 |
| GO Biological Processes | GO:0072577 | endothelial cell apoptotic process | -4.10 | 4/67 |
| GO Biological Processes | GO:2000353 | positive regulation of endothelial cell apoptotic process | -4.09 | 3/25 |
| GO Biological Processes | GO:1904037 | positive regulation of epithelial cell apoptotic process | -3.53 | 3/38 |
| GO Biological Processes | GO:0051271 | negative regulation of cellular component movement | -2.67 | 6/390 |
| GO Biological Processes | GO:0002696 | positive regulation of leukocyte activation | -2.67 | 6/391 |
| GO Biological Processes | GO:0050678 | regulation of epithelial cell proliferation | -2.64 | 6/396 |
| GO Biological Processes | GO:0001937 | negative regulation of endothelial cell proliferation | -2.61 | 3/79 |
| GO Biological Processes | GO:0050867 | positive regulation of cell activation | -2.59 | 6/406 |
| GO Biological Processes | GO:0030155 | regulation of cell adhesion | -2.53 | 8/716 |
| GO Biological Processes | GO:0010942 | positive regulation of cell death | -2.44 | 8/739 |
| GO Biological Processes | GO:1903037 | regulation of leukocyte cell-cell adhesion | -2.36 | 5/315 |
| GO Biological Processes | GO:0050673 | epithelial cell proliferation | -2.34 | 6/455 |
| GO Biological Processes | GO:0050870 | positive regulation of T cell activation | -2.27 | 4/206 |
| GO Biological Processes | GO:0006935 | chemotaxis | -2.18 | 7/649 |
| GO Biological Processes | GO:0042330 | taxis | -2.18 | 7/651 |
| GO Biological Processes | GO:0007159 | leukocyte cell-cell adhesion | -2.17 | 5/349 |
| GO Biological Processes | GO:0030595 | leukocyte chemotaxis | -2.15 | 4/223 |
| GO Biological Processes | GO:2000146 | negative regulation of cell motility | -2.14 | 5/356 |
| GO Biological Processes | GO:0050900 | leukocyte migration | -2.13 | 6/504 |
| GO Biological Processes | GO:1903039 | positive regulation of leukocyte cell-cell adhesion | -2.13 | 4/226 |
| GO Biological Processes | GO:0071621 | granulocyte chemotaxis | -2.11 | 3/119 |
| GO Biological Processes | GO:0043065 | positive regulation of apoptotic process | -2.08 | 7/679 |
| GO Biological Processes | GO:0051928 | positive regulation of calcium ion transport | -2.08 | 3/122 |
| GO Biological Processes | GO:0043068 | positive regulation of programmed cell death | -2.05 | 7/688 |
| GO Biological Processes | GO:0045862 | positive regulation of proteolysis | -4.43 | 8/370 |
| GO Biological Processes | GO:0052547 | regulation of peptidase activity | -3.77 | 8/460 |
| GO Biological Processes | GO:0051345 | positive regulation of hydrolase activity | -3.53 | 10/777 |
| GO Biological Processes | GO:0010952 | positive regulation of peptidase activity | -3.22 | 5/200 |
| GO Biological Processes | GO:0035265 | organ growth | -4.16 | 6/204 |
| GO Biological Processes | GO:0061384 | heart trabecula morphogenesis | -3.68 | 3/34 |
| GO Biological Processes | GO:0048729 | tissue morphogenesis | -3.31 | 9/680 |
| GO Biological Processes | GO:0061383 | trabecula morphogenesis | -3.21 | 3/49 |
| GO Biological Processes | GO:0048589 | developmental growth | -2.67 | 8/680 |
| GO Biological Processes | GO:0055024 | regulation of cardiac muscle tissue development | -2.31 | 3/101 |
| GO Biological Processes | GO:0055017 | cardiac muscle tissue growth | -2.25 | 3/106 |
| GO Biological Processes | GO:0060419 | heart growth | -2.17 | 3/113 |
| GO Biological Processes | GO:0048738 | cardiac muscle tissue development | -2.08 | 4/234 |
| GO Biological Processes | GO:0033002 | muscle cell proliferation | -2.04 | 4/240 |
| GO Biological Processes | GO:0042063 | gliogenesis | -4.13 | 7/301 |
| GO Biological Processes | GO:0008347 | glial cell migration | -3.11 | 3/53 |
| GO Biological Processes | GO:0048708 | astrocyte differentiation | -2.56 | 3/82 |
| GO Biological Processes | GO:0010001 | glial cell differentiation | -2.14 | 4/225 |
| GO Biological Processes | GO:0018108 | peptidyl-tyrosine phosphorylation | -2.06 | 5/371 |
| GO Biological Processes | GO:0018212 | peptidyl-tyrosine modification | -2.05 | 5/374 |
| KEGG Pathway | hsa03320 | PPAR signaling pathway | -3.97 | 4/72 |
| GO Biological Processes | GO:0001101 | response to acid chemical | -3.75 | 7/347 |
| GO Biological Processes | GO:0072001 | renal system development | -2.50 | 5/292 |
| GO Biological Processes | GO:0001655 | urogenital system development | -2.27 | 5/330 |
| GO Biological Processes | GO:0032526 | response to retinoic acid | -2.21 | 3/109 |
| GO Biological Processes | GO:1901800 | positive regulation of proteasomal protein catabolic process | -3.31 | 4/107 |
| GO Biological Processes | GO:0032103 | positive regulation of response to external stimulus | -3.08 | 6/325 |
| GO Biological Processes | GO:1903052 | positive regulation of proteolysis involved in cellular protein catabolic process | -3.07 | 4/124 |
| GO Biological Processes | GO:1903050 | regulation of proteolysis involved in cellular protein catabolic process | -3.03 | 5/220 |
| GO Biological Processes | GO:1903364 | positive regulation of cellular protein catabolic process | -2.82 | 4/145 |
| GO Biological Processes | GO:1903362 | regulation of cellular protein catabolic process | -2.77 | 5/253 |
| GO Biological Processes | GO:0050729 | positive regulation of inflammatory response | -2.75 | 4/151 |
| GO Biological Processes | GO:0061136 | regulation of proteasomal protein catabolic process | -2.41 | 4/188 |
| GO Biological Processes | GO:0045732 | positive regulation of protein catabolic process | -2.17 | 4/221 |
| GO Biological Processes | GO:0002526 | acute inflammatory response | -2.16 | 4/222 |
| GO Biological Processes | GO:0050727 | regulation of inflammatory response | -2.12 | 6/508 |
| GO Biological Processes | GO:0031349 | positive regulation of defense response | -2.08 | 6/517 |
| GO Biological Processes | GO:0031331 | positive regulation of cellular catabolic process | -2.06 | 5/372 |
| GO Biological Processes | GO:1901654 | response to ketone | -3.24 | 5/198 |
| GO Biological Processes | GO:0043393 | regulation of protein binding | -3.10 | 5/213 |
| Canonical Pathways | M145 | PID P53 DOWNSTREAM PATHWAY | -2.91 | 4/137 |
| GO Biological Processes | GO:0007178 | transmembrane receptor protein serine/threonine kinase signaling pathway | -2.87 | 6/356 |
| GO Biological Processes | GO:0001666 | response to hypoxia | -2.86 | 6/358 |
| GO Biological Processes | GO:0010038 | response to metal ion | -2.83 | 6/363 |
| GO Biological Processes | GO:0036293 | response to decreased oxygen levels | -2.79 | 6/370 |
| GO Biological Processes | GO:0070482 | response to oxygen levels | -2.65 | 6/394 |
| GO Biological Processes | GO:0090288 | negative regulation of cellular response to growth factor stimulus | -2.59 | 4/168 |
| GO Biological Processes | GO:0048732 | gland development | -2.40 | 6/442 |
| GO Biological Processes | GO:0007179 | transforming growth factor beta receptor signaling pathway | -2.34 | 4/197 |
| GO Biological Processes | GO:0032091 | negative regulation of protein binding | -2.24 | 3/107 |
| GO Biological Processes | GO:0017015 | regulation of transforming growth factor beta receptor signaling pathway | -2.10 | 3/120 |
| GO Biological Processes | GO:1903844 | regulation of cellular response to transforming growth factor beta stimulus | -2.08 | 3/122 |
| GO Biological Processes | GO:0051098 | regulation of binding | -2.07 | 5/370 |
| GO Biological Processes | GO:0090101 | negative regulation of transmembrane receptor protein serine/threonine kinase signaling pathway | -2.04 | 3/126 |
| GO Biological Processes | GO:1901652 | response to peptide | -2.02 | 6/534 |
| GO Biological Processes | GO:0045104 | intermediate filament cytoskeleton organization | -3.21 | 3/49 |
| GO Biological Processes | GO:0045103 | intermediate filament-based process | -3.18 | 3/50 |
| GO Biological Processes | GO:0043010 | camera-type eye development | -2.38 | 5/311 |
| GO Biological Processes | GO:0001654 | eye development | -2.11 | 5/361 |
| GO Biological Processes | GO:0150063 | visual system development | -2.09 | 5/365 |
| GO Biological Processes | GO:0048880 | sensory system development | -2.07 | 5/370 |
| GO Biological Processes | GO:0030278 | regulation of ossification | -3.13 | 5/209 |
| GO Biological Processes | GO:0030500 | regulation of bone mineralization | -2.65 | 3/76 |
| GO Biological Processes | GO:0001503 | ossification | -2.57 | 6/408 |
| GO Biological Processes | GO:0070167 | regulation of biomineral tissue development | -2.39 | 3/94 |
| GO Biological Processes | GO:0030282 | bone mineralization | -2.17 | 3/113 |
| GO Biological Processes | GO:1901214 | regulation of neuron death | -3.12 | 6/318 |
| GO Biological Processes | GO:0070997 | neuron death | -2.88 | 6/355 |
| GO Biological Processes | GO:1901215 | negative regulation of neuron death | -2.24 | 4/210 |
| GO Biological Processes | GO:0043523 | regulation of neuron apoptotic process | -2.22 | 4/213 |
| GO Biological Processes | GO:0051402 | neuron apoptotic process | -2.02 | 4/243 |
| GO Biological Processes | GO:0071222 | cellular response to lipopolysaccharide | -3.11 | 5/212 |
| GO Biological Processes | GO:0071219 | cellular response to molecule of bacterial origin | -2.99 | 5/225 |
| GO Biological Processes | GO:0071216 | cellular response to biotic stimulus | -2.80 | 5/249 |
| GO Biological Processes | GO:0009617 | response to bacterium | -2.48 | 8/729 |
| Reactome Gene Sets | R-HSA-449147 | Signaling by Interleukins | -2.32 | 6/461 |
| GO Biological Processes | GO:0030029 | actin filament-based process | -2.29 | 8/785 |
| GO Biological Processes | GO:0032496 | response to lipopolysaccharide | -2.23 | 5/338 |
| Reactome Gene Sets | R-HSA-6785807 | Interleukin-4 and Interleukin-13 signaling | -2.22 | 3/108 |
| GO Biological Processes | GO:0002237 | response to molecule of bacterial origin | -2.13 | 5/358 |
| GO Biological Processes | GO:0070252 | actin-mediated cell contraction | -2.12 | 3/118 |
| GO Biological Processes | GO:0001706 | endoderm formation | -3.08 | 3/54 |
| GO Biological Processes | GO:0001704 | formation of primary germ layer | -3.05 | 4/126 |
| GO Biological Processes | GO:0016055 | Wnt signaling pathway | -2.69 | 7/525 |
| GO Biological Processes | GO:0198738 | cell-cell signaling by wnt | -2.68 | 7/527 |
| GO Biological Processes | GO:0007492 | endoderm development | -2.62 | 3/78 |
| GO Biological Processes | GO:0060828 | regulation of canonical Wnt signaling pathway | -2.48 | 5/294 |
| GO Biological Processes | GO:0007369 | gastrulation | -2.41 | 4/188 |
| GO Biological Processes | GO:1905114 | cell surface receptor signaling pathway involved in cell-cell signaling | -2.25 | 7/631 |
| GO Biological Processes | GO:0060070 | canonical Wnt signaling pathway | -2.22 | 5/340 |
| GO Biological Processes | GO:0030111 | regulation of Wnt signaling pathway | -2.04 | 5/376 |
| GO Biological Processes | GO:0007420 | brain development | -3.04 | 9/741 |
| GO Biological Processes | GO:0060322 | head development | -2.87 | 9/784 |
| GO Biological Processes | GO:0046394 | carboxylic acid biosynthetic process | -3.00 | 7/462 |
| GO Biological Processes | GO:0016053 | organic acid biosynthetic process | -3.00 | 7/463 |
| GO Biological Processes | GO:1901607 | alpha-amino acid biosynthetic process | -2.85 | 3/65 |
| GO Biological Processes | GO:0008652 | cellular amino acid biosynthetic process | -2.55 | 3/83 |
| GO Biological Processes | GO:0043408 | regulation of MAPK cascade | -2.99 | 9/754 |
| GO Biological Processes | GO:0043405 | regulation of MAP kinase activity | -2.95 | 6/344 |
| GO Biological Processes | GO:0051129 | negative regulation of cellular component organization | -2.95 | 9/764 |
| GO Biological Processes | GO:0043406 | positive regulation of MAP kinase activity | -2.69 | 5/263 |
| GO Biological Processes | GO:0071902 | positive regulation of protein serine/threonine kinase activity | -2.20 | 5/343 |
| GO Biological Processes | GO:0071900 | regulation of protein serine/threonine kinase activity | -2.04 | 6/528 |
| GO Biological Processes | GO:1901361 | organic cyclic compound catabolic process | -2.93 | 9/768 |
| GO Biological Processes | GO:0006066 | alcohol metabolic process | -2.05 | 5/374 |
| GO Biological Processes | GO:0007612 | learning | -2.83 | 4/144 |
| GO Biological Processes | GO:0007611 | learning or memory | -2.73 | 5/258 |
| GO Biological Processes | GO:0007268 | chemical synaptic transmission | -2.51 | 8/720 |
| GO Biological Processes | GO:0098916 | anterograde trans-synaptic signaling | -2.51 | 8/720 |
| GO Biological Processes | GO:0099537 | trans-synaptic signaling | -2.48 | 8/728 |
| GO Biological Processes | GO:0099536 | synaptic signaling | -2.47 | 8/733 |
| GO Biological Processes | GO:0050890 | cognition | -2.47 | 5/297 |
| GO Biological Processes | GO:0050808 | synapse organization | -2.46 | 6/431 |
| GO Biological Processes | GO:0050804 | modulation of chemical synaptic transmission | -2.37 | 6/450 |
| GO Biological Processes | GO:0099177 | regulation of trans-synaptic signaling | -2.36 | 6/451 |
| GO Biological Processes | GO:0007610 | behavior | -2.30 | 7/617 |

**Supplementary Table 3.** Cancer-related, chemotherapy-related and immune-related biological processes that CSPG4 might regulate through Gene set enrichment analysis (GSEA).

| Type | Description | Set Size | Enrichment Score | NES | P-Value | adjusted P-value | Q-Value | Rank | Leading_edge |
| --- | --- | --- | --- | --- | --- | --- | --- | --- | --- |
| Cancer-related | KEGG_BLADDER_CANCER | 130 | 0.71 | 3.10 | 2.24E-03 | 7.33E-03 | 3.79E-03 | 5264 | tags=52%, list=14%, signal=45% |
| Cancer-related | KEGG_PATHWAYS_IN_CANCER | 270 | 0.63 | 2.99 | 2.27E-03 | 7.33E-03 | 3.79E-03 | 5561 | tags=53%, list=15%, signal=45% |
| Cancer-related | KEGG_TGF_BETA_SIGNALING_PATHWAY | 90 | 0.72 | 2.92 | 2.37E-03 | 7.33E-03 | 3.79E-03 | 4748 | tags=49%, list=13%, signal=43% |
| Cancer-related | KEGG_VASCULAR_SMOOTH_MUSCLE_CONTRACTION | 129 | 0.67 | 2.89 | 2.27E-03 | 7.33E-03 | 3.79E-03 | 6524 | tags=47%, list=18%, signal=39% |
| Chemotherapy-related | KEGG_DRUG_METABOLISM_CYTOCHROME_P450 | 83 | 0.82 | 3.28 | 2.31E-03 | 7.33E-03 | 3.79E-03 | 4766 | tags=75%, list=13%, signal=65% |
| Chemotherapy-related | KEGG_DRUG_METABOLISM_OTHER_ENZYMES | 199 | 0.70 | 3.22 | 2.23E-03 | 7.33E-03 | 3.79E-03 | 6553 | tags=56%, list=18%, signal=46% |
| Immune-related | KEGG_B_CELL_RECEPTOR_SIGNALING_PATHWAY | 177 | 0.64 | 2.86 | 2.29E-03 | 7.33E-03 | 3.79E-03 | 6595 | tags=55%, list=18%, signal=46% |
| Immune-related | KEGG_LEUKOCYTE_TRANSENDOTHELIAL_MIGRATION | 325 | 0.59 | 2.84 | 2.34E-03 | 7.33E-03 | 3.79E-03 | 8543 | tags=51%, list=23%, signal=39% |
| Immune-related | KEGG_NATURAL_KILLER_CELL_MEDIATED_CYTOTOXICITY | 74 | 0.71 | 2.81 | 2.32E-03 | 7.33E-03 | 3.79E-03 | 4748 | tags=49%, list=13%, signal=42% |
| Immune-related | KEGG_T_CELL_RECEPTOR_SIGNALING_PATHWAY | 83 | 0.70 | 2.81 | 2.31E-03 | 7.33E-03 | 3.79E-03 | 4748 | tags=46%, list=13%, signal=40% |

**Supplementary Table 4.** Cancer-related, chemotherapy-related and immune-related KEGG pathways that CSPG4 might regulate through Gene set enrichment analysis (GSEA).

| Type | Description | Set Size | Enrichment Score | NES | P-Value | adjusted P-value | Q-Value | Rank | Leading_edge |
| --- | --- | --- | --- | --- | --- | --- | --- | --- | --- |
| Cancer-related | GO_ACTIVATION_OF_MAPKK_ACTIVITY | 52 | 0.56 | 2.05 | 4.75E-03 | 1.15E-02 | 4.90E-03 | 6305 | tags=29%, list=17%, signal=24% |
| Cancer-related | GO_TRNA_METHYLATION | 40 | -0.39 | -1.30 | 1.13E-01 | 1.71E-01 | 7.27E-02 | 19308 | tags=68%, list=53%, signal=32% |
| Cancer-related | GO_TRNA_MODIFICATION | 88 | -0.31 | -1.24 | 1.50E-01 | 2.19E-01 | 9.31E-02 | 19550 | tags=60%, list=53%, signal=28% |
| Chemotherapy-related | GO_RESPONSE_TO_DRUG | 393 | 0.44 | 2.20 | 2.33E-03 | 6.39E-03 | 2.72E-03 | 7467 | tags=34%, list=20%, signal=27% |
| Chemotherapy-related | GO_CELLULAR_RESPONSE_TO_DRUG | 70 | 0.55 | 2.13 | 2.41E-03 | 6.39E-03 | 2.72E-03 | 7467 | tags=41%, list=20%, signal=33% |
| Chemotherapy-related | GO_CHEMICAL_SYNAPTIC_TRANSMISSION_POSTSYNAPTIC | 111 | 0.71 | 3.01 | 2.32E-03 | 6.39E-03 | 2.72E-03 | 7388 | tags=69%, list=20%, signal=56% |
| Chemotherapy-related | GO_REGULATION_OF_SYSTEMIC_ARTERIAL_BLOOD_PRESSURE_MEDIATED_BY_A_CHEMICAL_SIGNAL | 50 | 0.68 | 2.49 | 2.35E-03 | 6.39E-03 | 2.72E-03 | 5245 | tags=56%, list=14%, signal=48% |
| Chemotherapy-related | GO_RESPONSE_TO_ACID_CHEMICAL | 130 | 0.57 | 2.51 | 2.35E-03 | 6.39E-03 | 2.72E-03 | 6209 | tags=38%, list=17%, signal=31% |
| Chemotherapy-related | GO_CELLULAR_RESPONSE_TO_CHEMICAL_STRESS | 346 | 0.39 | 1.88 | 2.36E-03 | 6.39E-03 | 2.72E-03 | 7235 | tags=28%, list=20%, signal=23% |
| Chemotherapy-related | GO_CELLULAR_RESPONSE_TO_ACID_CHEMICAL | 74 | 0.62 | 2.43 | 2.36E-03 | 6.39E-03 | 2.72E-03 | 7504 | tags=49%, list=21%, signal=39% |
| Chemotherapy-related | GO_PRESYNAPTIC_MODULATION_OF_CHEMICAL_SYNAPTIC_TRANSMISSION | 15 | 0.69 | 1.89 | 6.86E-03 | 1.60E-02 | 6.80E-03 | 5364 | tags=47%, list=15%, signal=40% |
| Chemotherapy-related | GO_ESTABLISHMENT_OR_MAINTENANCE_OF_TRANSMEMBRANE_ELECTROCHEMICAL_GRADIENT | 14 | 0.67 | 1.80 | 6.88E-03 | 1.60E-02 | 6.80E-03 | 3913 | tags=36%, list=11%, signal=32% |
| Chemotherapy-related | GO_CHEMICAL_HOMEOSTASIS_WITHIN_A_TISSUE | 11 | 0.66 | 1.66 | 3.68E-02 | 6.42E-02 | 2.73E-02 | 4006 | tags=45%, list=11%, signal=40% |
| Chemotherapy-related | GO_POSITIVE_REGULATION_OF_TRANSCRIPTION_FROM_RNA_POLYMERASE_II_PROMOTER_INVOLVED_IN_CELLULAR_RESPONSE_TO_CHEMICAL_STIMULUS | 22 | 0.43 | 1.30 | 1.76E-01 | 2.51E-01 | 1.07E-01 | 11360 | tags=55%, list=31%, signal=38% |
| Immune-related | GO_CYTOKINE_PRODUCTION_INVOLVED_IN_IMMUNE_RESPONSE | 104 | 0.54 | 2.28 | 2.30E-03 | 6.39E-03 | 2.72E-03 | 7232 | tags=45%, list=20%, signal=36% |
| Immune-related | GO_NEGATIVE_REGULATION_OF_CYTOKINE_PRODUCTION_INVOLVED_IN_IMMUNE_RESPONSE | 24 | 0.67 | 2.04 | 2.34E-03 | 6.39E-03 | 2.72E-03 | 7137 | tags=58%, list=20%, signal=47% |
| Immune-related | GO_POSITIVE_REGULATION_OF_HUMORAL_IMMUNE_RESPONSE | 19 | 0.68 | 1.98 | 2.35E-03 | 6.39E-03 | 2.72E-03 | 6756 | tags=63%, list=18%, signal=52% |
| Immune-related | GO_POSITIVE_REGULATION_OF_PRODUCTION_OF_MOLECULAR_MEDIATOR_OF_IMMUNE_RESPONSE | 99 | 0.46 | 1.90 | 2.35E-03 | 6.39E-03 | 2.72E-03 | 7685 | tags=40%, list=21%, signal=32% |
| Immune-related | GO_PRODUCTION_OF_MOLECULAR_MEDIATOR_OF_IMMUNE_RESPONSE | 302 | 0.51 | 2.44 | 2.35E-03 | 6.39E-03 | 2.72E-03 | 7309 | tags=46%, list=20%, signal=37% |
| Immune-related | GO_POSITIVE_REGULATION_OF_ADAPTIVE_IMMUNE_RESPONSE | 108 | 0.49 | 2.05 | 2.35E-03 | 6.39E-03 | 2.72E-03 | 7685 | tags=39%, list=21%, signal=31% |
| Immune-related | GO_REGULATION_OF_INNATE_IMMUNE_RESPONSE | 303 | 0.35 | 1.68 | 2.35E-03 | 6.39E-03 | 2.72E-03 | 7323 | tags=26%, list=20%, signal=21% |
| Immune-related | GO_REGULATION_OF_PRODUCTION_OF_MOLECULAR_MEDIATOR_OF_IMMUNE_RESPONSE | 142 | 0.49 | 2.19 | 2.35E-03 | 6.39E-03 | 2.72E-03 | 7232 | tags=42%, list=20%, signal=33% |
| Immune-related | GO_NEGATIVE_REGULATION_OF_IMMUNE_EFFECTOR_PROCESS | 124 | 0.51 | 2.20 | 2.36E-03 | 6.39E-03 | 2.72E-03 | 7290 | tags=41%, list=20%, signal=33% |
| Immune-related | GO_HUMORAL_IMMUNE_RESPONSE_MEDIATED_BY_CIRCULATING_IMMUNOGLOBULIN | 143 | 0.70 | 3.10 | 2.36E-03 | 6.39E-03 | 2.72E-03 | 5835 | tags=67%, list=16%, signal=57% |
| Immune-related | GO_REGULATION_OF_HUMORAL_IMMUNE_RESPONSE | 132 | 0.70 | 3.08 | 2.36E-03 | 6.39E-03 | 2.72E-03 | 4782 | tags=59%, list=13%, signal=52% |
| Immune-related | GO_NEGATIVE_REGULATION_OF_LEUKOCYTE_APOPTOTIC_PROCESS | 49 | 0.58 | 2.09 | 2.39E-03 | 6.39E-03 | 2.72E-03 | 7381 | tags=51%, list=20%, signal=41% |
| Immune-related | GO_POSITIVE_REGULATION_OF_LEUKOCYTE_PROLIFERATION | 149 | 0.53 | 2.34 | 2.39E-03 | 6.39E-03 | 2.72E-03 | 8543 | tags=50%, list=23%, signal=39% |
| Immune-related | GO_POSITIVE_REGULATION_OF_MACROPHAGE_MIGRATION | 26 | 0.74 | 2.28 | 2.39E-03 | 6.39E-03 | 2.72E-03 | 3109 | tags=50%, list=9%, signal=46% |
| Immune-related | GO_T_CELL_RECEPTOR_SIGNALING_PATHWAY | 204 | 0.31 | 1.44 | 4.72E-03 | 1.15E-02 | 4.90E-03 | 9556 | tags=36%, list=26%, signal=27% |

|  | **CAV1** | | **COL6A2** | | **FABP4** | | **FBLN1** | | **PCOLCE** | |
| --- | --- | --- | --- | --- | --- | --- | --- | --- | --- | --- |
|  | NES | P | NES | P | NES | P | NES | P | NES | P |
| **Cancer-related KEGG pathways** |  |  |  |  |  |  |  |  |  |  |
| KEGG_PATHWAYS_IN_CANCER (hsa05200) | 1.895 | **0.001** | 1.656 | **0.001** | 1.139 | 0.141 | -1.470 | **0.002** | 1.767 | **0.001** |
| KEGG_BLADDER_CANCER (hsa05219) | 1.451 | **0.029** | 1.297 | 0.137 | -1.517 | **0.026** | -1.040 | 0.398 | 1.402 | 0.060 |
| **Immune-related KEGG pathways** |  |  |  |  |  |  |  |  |  |  |
| KEGG_T_CELL_RECEPTOR_SIGNALING_PATHWAY (hsa04660) | 1.826 | **0.002** | 1.724 | **0.001** | -0.811 | 0.908 | -1.780 | **0.002** | 1.649 | **0.001** |
| KEGG_B_CELL_RECEPTOR_SIGNALING_PATHWAY (hsa04662) | 1.717 | **0.002** | 1.696 | **0.001** | 1.864 | **0.003** | -1.420 | **0.033** | 1.723 | **0.001** |
| KEGG_NATURAL_KILLER_CELL_MEDIATED_CYTOTOXICITY (hsa04650) | 1.982 | **0.002** | 1.837 | **0.001** | 0.840 | 0.800 | -1.994 | **0.002** | 1.724 | **0.001** |
| **Chemotherapy-related KEGG pathways** |  |  |  |  |  |  |  |  |  |  |
| KEGG_DRUG_METABOLISM_CYTOCHROME_P450 (hsa00982) | -1.549 | **0.008** | -1.254 | 0.110 | -1.173 | 0.153 | 1.759 | **0.002** | -1.040 | 0.329 |
| KEGG_DRUG_METABOLISM_OTHER_ENZYMES (hsa00983) | 0.926 | 0.589 | -1.187 | 0.176 | -1.525 | **0.019** | -1.280 | 0.110 | -1.074 | 0.346 |

**Supplementary Table 5.** Cancer-related, chemotherapy-related and immune-related KEGG pathways that CAV1, COL6A2, FABP4, FBLN1 and PCOLCE might regulate through Gene set enrichment analysis (GSEA).

**Supplementary R Codes**

**R Code 1-GSVA.**

library(msigdbr)

library(dplyr)

library(data.table)

library(GSVA)

library(limma)

library(stringr)

library(ggplot2)

Sys.setenv(LANGUAGE = "en")

options(stringsAsFactors = FALSE)

# options(mc.cores = parallel::detectCores())

h <- msigdbr(species = "Homo sapiens",

category = "C2")

h <- select(h, gs_name, gene_symbol) %>%

as.data.frame %>%

split(., .$gs_name) %>%

lapply(., function(x)(x$gene_symbol))

gs <- lapply(h, unique)

#count <- table(unlist(gs))

#keep <- names(which(table(unlist(gs)) < 2))

#gs <- lapply(gs, function(x) intersect(keep, x))

gs <- gs[lapply(gs, length) > 0]

head(gs)

save(gs, file = "path1\\path2\\c2.gs.RData")

## GSVA

(load("path1\\path2\\c2.gs.RData"))

gsym.expr <- read.csv("path1\\path2\\file1.csv", row.names = 1)

head(gsym.expr)

gsva_es <- gsva(as.matrix(gsym.expr), gs)

head(gsva_es)

write.csv(gsva_es, "path1\\path2\\gsva_output.csv", quote = F)

**R Code 2-Limma.**

#source("http://bioconductor.org/biocLite.R")

#biocLite("limma")

logFoldChange=1

adjustP=0.05

library(limma)

setwd("path1\\path2")

rt=read.table("limma for GSE77883.txt",sep="\t",header=T,check.names=F)

rt=as.matrix(rt)

rownames(rt)=rt[,1]

exp=rt[,2:ncol(rt)]

dimnames=list(rownames(exp),colnames(exp))

rt=matrix(as.numeric(as.matrix(exp)),nrow=nrow(exp),dimnames=dimnames)

rt=avereps(rt)

rt=normalizeBetweenArrays(as.matrix(rt))

rt=log2(rt+1)

#differential

Type=c(rep("resistantT24",3),rep("untreatedT24",3))

design <- model.matrix(~0+factor(Type))

colnames(design) <- c("resistantT24","untreatedT24")

fit <- lmFit(rt,design)

cont.matrix<-makeContrasts(resistantT24-untreatedT24,levels=design)

fit2 <- contrasts.fit(fit, cont.matrix)

fit2 <- eBayes(fit2)

allDiff=topTable(fit2,adjust='fdr',number=200000)

write.table(allDiff,file="limmaTab.xls",sep="\t",quote=F)

type=sapply(strsplit(rownames(allDiff),"\\|"),"[",2)

protein=allDiff[type=="protein_coding",]

lncRNA=allDiff[type=="lncRNA",]

rownames(allDiff)=gsub("(.*?)\\|.*","\\1",rownames(allDiff))

rownames(protein)=gsub("(.*?)\\|.*","\\1",rownames(protein))

rownames(lncRNA)=gsub("(.*?)\\|.*","\\1",rownames(lncRNA))

rownames(rt)=gsub("(.*?)\\|.*","\\1",rownames(rt))

#write protein and lncRNA table

diffSig <- allDiff[with(allDiff, (abs(logFC)>logFoldChange & adj.P.Val < adjustP )), ]

write.table(diffSig,file="diff.xls",sep="\t",quote=F)

#write expression level of diff gene

hmExp=rt[rownames(diffSig),]

diffExp=rbind(id=colnames(hmExp),hmExp)

write.table(diffExp,file="heatmap.txt",sep="\t",quote=F,col.names=F)

#write lncRNA table

lncRNASig <- lncRNA[with(lncRNA, (abs(logFC)>logFoldChange & adj.P.Val < adjustP )), ]

write.table(lncRNASig,file="lncRNA_diff.xls",sep="\t",quote=F)

#write expression level of diff lncRNA gene

hmExp=rt[rownames(lncRNASig),]

diffExp=rbind(id=colnames(hmExp),hmExp)

write.table(diffExp,file="lncRNA_heatmap.txt",sep="\t",quote=F,col.names=F)

#write protein table

proteinSig <- protein[with(protein, (abs(logFC)>logFoldChange & adj.P.Val < adjustP )), ]

write.table(proteinSig,file="protein_diff.xls",sep="\t",quote=F)

#write expression level of diff gene

hmExp=rt[rownames(proteinSig),]

diffExp=rbind(id=colnames(hmExp),hmExp)

write.table(diffExp,file="protein_heatmap.txt",sep="\t",quote=F,col.names=F)

#volcano

tiff(file="vol.tiff",

width = 12,

height =12,

units ="cm",

compression="lzw",

bg="white",

res=600)

xMax=max(-log10(allDiff$adj.P.Val))

yMax=max(abs(allDiff$logFC))

plot(-log10(allDiff$adj.P.Val), allDiff$logFC, xlab="-log10(adj.P.Val)",ylab="logFC",

main="Volcano", xlim=c(0,xMax),ylim=c(-yMax,yMax),yaxs="i",pch=20, cex=0.8)

diffSub=subset(allDiff, adj.P.Val<adjustP & logFC>logFoldChange)

points(-log10(diffSub$adj.P.Val), diffSub$logFC, pch=20, col="red",cex=0.8)

diffSub=subset(allDiff, adj.P.Val<adjustP & logFC<(-logFoldChange))

points(-log10(diffSub$adj.P.Val), diffSub$logFC, pch=20, col="green",cex=0.8)

abline(h=0,lty=2,lwd=3)

dev.off()

**R Code 3-pheatmap.**

setwd("path1\\path2")

rt=read.table("heatmap for GSE77883.txt",sep="\t",header=T,row.names=1,check.names=F)

rt=t(scale(t(rt)))

library(pheatmap)

Type=c(rep("Untreated T24 cells",3),rep("GEM-resistant T24 cells",3))

fac=levels(factor(Type))

rt1=rt[,Type==fac[1]]

rt2=rt[,Type==fac[2]]

rt=cbind(rt1,rt2)

Type=c(rep(as.character(fac[1]),ncol(rt1)),rep(as.character(fac[2]),ncol(rt2)))

names(Type)=colnames(rt)

Type=as.data.frame(Type)

tiff(file="heatmap.tiff",

width = 13,

height =10,

units ="cm",

compression="lzw",

bg="white",

res=600)

pheatmap(rt,

annotation=Type,

#color = colorRampPalette(c("green", "black", "red"))(50),

cluster_cols =F,

fontsize = 8,

fontsize_row=6,

show_rownames=F,

show_colnames=F,treeheight_row = 10,

fontsize_col=8)

**R Code 4-clusterProfiler for GO.**

setwd("path1\\path2")

library("clusterProfiler")

library("org.Hs.eg.db")

rt=read.table("id.txt",sep="\t",header=T,check.names=F)

rt=rt[is.na(rt[,"entrezID"])==F,]

geneFC=rt$logFC

gene=rt$entrezID

names(geneFC)=gene

########################BP##################

kk <- enrichGO(gene = gene,OrgDb = org.Hs.eg.db,ont="BP",pvalueCutoff =0.05, qvalueCutoff = 0.05)

write.table(kk,file="GOBP.txt",sep="\t",quote=F,row.names = F)

tiff(file="barplotBP.tiff",width = 20,height = 20,units ="cm",compression="lzw",bg="white",res=600)

barplot(kk, drop = TRUE, showCategory = 10)

dev.off()

tiff(file="dotplotBP.tiff",width = 20,height = 20,units ="cm",compression="lzw",bg="white",res=600)

dotplot(kk,showCategory = 10)

dev.off()

#############################CC##############

kk <- enrichGO(gene = gene,OrgDb = org.Hs.eg.db,ont="CC",pvalueCutoff =0.05, qvalueCutoff = 0.05)

write.table(kk,file="GOCC.txt",sep="\t",quote=F,row.names = F)

tiff(file="barplotCC.tiff",width = 20,height = 20,units ="cm",compression="lzw",bg="white",res=600)

barplot(kk, drop = TRUE, showCategory = 10)

dev.off()

tiff(file="dotplotCC.tiff",width = 20,height = 20,units ="cm",compression="lzw",bg="white",res=600)

dotplot(kk,showCategory = 10)

dev.off()

#############################MF##############

kk <- enrichGO(gene = gene,OrgDb = org.Hs.eg.db,ont="MF",pvalueCutoff =0.05, qvalueCutoff = 0.05)

write.table(kk,file="GOMF.txt",sep="\t",quote=F,row.names = F)

tiff(file="barplotMF.tiff",width = 20,height = 20,units ="cm",compression="lzw",bg="white",res=600)

barplot(kk, drop = TRUE, showCategory = 10)

dev.off()

tiff(file="dotplotMF.tiff",width = 20,height = 20,units ="cm",compression="lzw",bg="white",res=600)

dotplot(kk,showCategory = 10)

dev.off()

**R Code 5-clusterProfiler for KEGG.**

setwd("path1\\path2")

library("clusterProfiler")

rt=read.table("id.txt",sep="\t",header=T,check.names=F)

rt=rt[is.na(rt[,"entrezID"])==F,]

geneFC=rt$logFC

gene=rt$entrezID

names(geneFC)=gene

kk <- enrichKEGG(gene = gene, organism = "hsa", pvalueCutoff =0.05, qvalueCutoff =0.05)

write.table(kk,file="KEGG.txt",sep="\t",quote=F,row.names = F)

cnetplot(kk, categorySize="pvalue", foldChange=geneFC)

tiff(file="barplot.tiff",width = 13,height = 5,units ="cm",compression="lzw",bg="white",res=600)

barplot(kk, drop = TRUE, showCategory = 100)

dev.off()

tiff(file="dotplot.tiff",width = 15,height = 9,units ="cm",compression="lzw",bg="white",res=600)

dotplot(kk, showCategory = 100)

dev.off()

browseKEGG(kk, "hsa03320")

library("pathview")

keggxls=read.table("KEGG.txt",sep="\t",header=T)

for(i in keggxls$ID){

pv.out <- pathview(gene.data = geneFC, pathway.id = i, species = "hsa", out.suffix = "pathview")

}
